# Herbal Honey Preparations of Curcuma Xanthorriza and Black Cumin Protect against Carcinogenesis through Antioxidant and Immunomodulatory Activities in Sprague Dawley (SD) Rats Induced with Dimethylbenz(a)anthracene

**DOI:** 10.3390/nu15020371

**Published:** 2023-01-11

**Authors:** Titiek Hidayati, Indrayanti Indrayanti, Endang Darmawan, Akrom Akrom

**Affiliations:** 1 Department of Public Health and Family Medicine, Faculty of Medicine and Health Science, Universitas Muhammadiyah Yogyakarta, Yogyakarta 55252, Indonesia; 2 Department of Anatomical Pathology, Faculty of Medicine and Health Science, Universitas Muhammadiyah Yogyakarta, Yogyakarta 55252, Indonesia; 3 Department of Pharmacology and Clinical Pharmacy, Faculty of Pharmacy, Universitas Ahmad Dahlan, Yogyakarta 55252, Indonesia; 4 Department of Pharmacology and Clinical Pharmacy, Master Pharmacy Degree Program, Faculty of Pharmacy, Universitas Ahmad Dahlan, Yogyakarta 55252, Indonesia; 5 Ahmad Dahlan Drug Information and Research Center, Universitas Ahmad Dahlan, Yogyakarta 55252, Indonesia

**Keywords:** herbal honey, curcuma xanthorriza, black cumin seed, chemopreventive, antioxidants, immunomodulatory

## Abstract

Background: Traditionally, Curcuma xanthorriza (CX), black cumin seed (BC), and honey have been used by the Indonesian people as medicinal ingredients to treat various health symptoms. CX extracts and BC have been proven in the laboratory as chemopreventive agents, antioxidants, and immunomodulators. In this study, we developed CX extract, BC oil, and honey into herbal honey preparations (CXBCH) and hypothesized that the preparations show chemopreventive activity. The purpose of the study was to determine the CXBCH potential as chemopreventive, antioxidant, and immunomodulatory. Method: In this experimental laboratory research, antioxidant, immunomodulatory, and cytotoxic activities were tested on human mammary cancer cell lines (T47D cells) while the chemopreventive activity of the CXBCH preparations on Sprague Dawley (SD) rats induced with dimethylbenzene(a)anthracene (DMBA). Results: CXBCH preparations demonstrated immunomodulatory, antioxidant, and cytotoxic activities in T47D, Hela, and HTB-183 cells and in DMBA-induced SD rats, as the preparations inhibited tumor nodule formation, increased the number of CD4, CD8 and CD4CD25 cells, and glutathione-S-transferase (GST) activity, and decreased serum NO levels. Conclusions: CXBCH preparations display chemopreventive, antioxidant, and immunomodulatory properties.

## 1. Introduction

Polycyclic aromatic hydrocarbons (PAH) 7,12 dimethylbenzene(a)anthracene (DMBA) is an air pollutant [1]. PAH compounds are one of the triggers for the increase in the incidence of breast cancer [2], lung cancer [3], and skin cancer [4]. Cigarette smoke and vehicle engine fumes are the primary sources of PAHs. Currently, smokers in Indonesia are estimated at more than 33% [5]. Indonesian urban communities are estimated to breathe polluted air daily with PAH content equivalent to PAH content in seven cigarettes, even though they are not active smokers [6]. The content of PAH compounds in smokeless tobacco ranges from 0.1–90 ng/g [7,8], while PAH has been found in a high concentration in tobacco cigarettes (40–100 ng per cigarette) [9,10,11]. Furthermore, it has been established that cigarettes contain 30–80 ng of benzanthracene molecules, whereas such compounds range from 25 to 280 ng for every gram of motor vehicle dust [12]. According to research conducted in a Japanese city, there are 0–83 picograms of benzanthracene compounds and 11–1923 picograms of total PAHs in per cubic meter of air [13,14]. Although there has not been any research on this subject up to this point, it is assumed that Indonesia has significant amounts of PAHs and benzanthracene in its urban air due to the country’s high population of smokers and motorized vehicles [15].

DMBA is converted to DMBA-3,4-diol-1,2-epoxide by cytochrome P450 enzymes 1A1 or 1B1 (CYP1A1 or CYP1B1) and microsomal hydrolase enzymes (DMBA-DE) [16]. Microsomal epoxide hydrolase (mEH) converts DMBA-3,4-epoxide to DMBA-3,4-dihydrodiol (DMBA-3,4-diol); subsequently, CYP1A1 or CYP1B1 oxidizes DMBA-3,4-diol to its ultimate carcinogenic form, DMBA-3,4-diol-1,2-epoxide [17]. DMBA-DE is a genotoxic [18] and immunosuppressive active metabolite of DMBA [18]. DMBA exposure and cigarette smoke are associated with increased serum nitric oxide (NO) levels [19]. In physiological processes including cell signaling and inflammatory management, nitric oxide (NO) molecules are vital [20]. At the same time, NO in excessive amounts will be oxidative, reactive, and genotoxic [21]. DMBA exposure is also associated with decreased immune response [22]. Administration for five days of DMBA 50 and 150 mg/kg BW in C57BL/6N wild type (WT) mice caused a decrease in spleen weight, lymphocyte count, and T lymphocyte proliferative activity, and suppressed bone marrow activity. Spleen weight, total lymphocyte count, and T lymphocyte proliferative activity were inversely related to the levels of DMBA metabolites in spleen tissues. DMBA levels in the blood are associated with the suppression of lymphoid tissues (colony forming unit (CFU)-preB) and myeloid tissues (CFU-GM) [16,23]. DMBA levels also reduce the number of bone marrow lymphoid cells and blood lymphocytes [16]. DMBA induction inhibits iNOS expression, NO secretion, and IL-12 secretion by macrophages [24]. CD4 and CD8 T lymphocytes maintain adaptive cellular immune responses against neoplasms [25]. DMBA induction has decreased the number of lymphocytes and the immune response [17]. The decrease in the number of lymphocytes and the immune response is thought to be one of the mechanisms for the development of genetic stress into neoplasms and neoplasms into tumor tissues or cancer [26]. More people, including children and pregnant women, are exposed to carcinogenic compounds, as the number of smokers and PAH pollutants in the air rises with the growing number of motor vehicles. This condition has increased the incidence of cancer in Indonesia [6,27].

The elimination of DMBA-DE from the body is accelerated in the presence of the antioxidant enzyme glutathione-S-transferase (GST) [28]. The GST enzyme conjugates with DMBA-DE into a water-soluble compound that makes it easy to be excreted [29]. Accelerated elimination of DMBA-DE is associated with detoxification, immunoprotection, and inhibition of carcinogenesis [30]. Antioxidant agents and herbal immunostimulants are believed to be able to inhibit the conversion of DMBA into DMBA-DE, and the decrease in lymphocytes and immunological responses due to DMBA exposure are believed to be effective in avoiding carcinogenesis [31]. Curcuma xanthorriza (CX) and black cumin seed (BC) (Nigella sativa) have been shown to exhibit immunomodulatory, antioxidant, and chemopreventive properties [32,33]. Researchers have demonstrated the BC chemopreventive activity and thymoquinone via cytoprotective antioxidant effects by inhibiting the CYP gene (phase I) activity and increasing the GST gene (phase II) activity via the activation of Nrf2, thereby increasing the production of the GST enzyme [33]. The administration of thymoquinone at 50 mg/kg BW has been shown to increase the antioxidant capacity of test animals, as indicated by an increase in SOD enzyme levels and a decrease in lipid peroxide [25,26,34]. Thymoquinone can also act as a scavenger agent and neutralize free radicals to reduce DMBA-DE levels [35]. Thymoquinone has been proven to decrease DNA adduct formation and nodule formation in DMBA-induced SD rats [36]. Like BC, empirically, Curcuma xanthorriza (CX) has also been used by the people of Indonesia as an immune system booster [32], anti-inflammatory [37], and antioxidant [38]. Indonesian people traditionally use CX on children with eating difficulty as it can serve as an appetite enhancer [32]. The main active substances of CX are Xanthorrhizol, curcumin, and curcuminoids [39]. Xanthorrhizol has been shown to display antioxidant activity by suppressing lipid peroxidation and decreasing Reactive Oxygen Superfamily (ROS) production [40]. Curcumin, one of the main active ingredients of CX, has been shown to have anti-inflammatory effects, be safe to use, and be well tolerated [41]. Honey contains various active substances, and its sweet taste has been used as a mixture in various traditional medicines to enhance the taste [42,43]. CX and BC combination with the addition of honey is thought to have a synergistic effect, by increasing antioxidant and immunomodulatory activity [44]. The research team has developed herbal honey preparations consisting of black cumin seed and curcuma xanthorriza with honey as the solvent called “CXBC herbal honey preparations” or CXBCH. It is hypothesized that CXBCH has chemopreventive potential due to their antioxidant and immunomodulatory properties. This study aimed to determine the chemopreventive activity of CXBCH preparations in Sprague Dawley (SD) rats induced with DMBA.

## 2. Materials and Methods

### 2.1. Instruments and Materials

#### 2.1.1. Research Protocol, Test Materials, Positive Control, and Carcinogen

The research protocol was ethically reviewed and had received a clearance label from the research ethics committee of Ahmad Dahlan university (Number: 012204031). A mixture of honey, black cumin seed oil, and Curcuma xanthorriza (CX) extracts (CXE) were provided by a traditional herbal medicine industry certified by the Indonesian Food and Drug Supervisory Agency. We obtained Tragacanth (Sigma-Aldrich, Jakarta, Indonesia, cat: G1128) as an emulsifier in the manufacture of this herbal honey preparation from an authorized agent of Sigma-Aldrich in Yogyakarta, Indonesia. The test plants were determined by an expert, Subagus Wahyuono, Apt from the Department of Biology Pharmacy, Faculty of Pharmacy, Gadjah Mada University. The extraction and preparation of the test materials were carried out with qualitative and quantitative phytochemical analysis. The thymoquinone (2-isopropyl-5 methyl-1,4-benzoquinone) (Sigma-Aldrich, Jakarta, Indonesia, cat. 274666-5G) and tamoxifen citrate (Sigma-Aldrich, Jakarta, Indonesia, cat.: T0015000) used as the positive control were obtained from Sigma while the carcinogen used was 7,12 dimethylbenzene(a)anthracene (DMBA) (Sigma, Jakarta, Indonesia, cat. D3254), dissolved in corn oil at 100 mg/kg BW [45].

#### 2.1.2. Experimental Cells and Animals

A viability test was performed on cancer cells (T47D, Hela and HTB-183) [46,47] while the chemopreventive effectiveness test of CXBCH preparations was carried out on female Sprague Dawley (SD) rats. We obtained cancer cell line (T47D, Hela and HTB-183 cells) from the Cancer Chemoprevention Research center (CCRC), Gadjah Mada University, Yogyakarta, Indonesia. We used 80 female Sprague Dawley rats aged 24–30 days with an average weight of 80–120 g obtained from the Preclinical Experimental and Animal Development Unit (PEADU), Gadjah Mada University, Yogyakarta, Indonesia. PEADU is a unit at Gadjah Mada University dedicated to providing experimental animals. Before being used for the experiment, the animals were acclimatized to the experimental room and cages and grouped by the treatment they received. Food and drink were provided ad libitum.

We conducted a chemopreventive test of the DMBA chemical carcinogen model using female test animals in line with past findings. It has been demonstrated that DMBA causes cancer in female Sprague Dawley rats between four and six weeks of age [45,48]. The animals were kept in standardized climatic settings (22–28 °C, 60–70% relative humidity, and a 12-h cycle of darkness and light). They were kept in properly ventilated cages and given access to unlimited amounts of water as well as pelleted food (broiler-II, Japfa Comfeed Ltd., Yogyakarta, Indonesia). All animal experiments were conducted in accordance with the guidelines established by Universitas Ahmad Dahlan’s ethical research committee.

We provided standard feed to all groups of the test animals. Japfa Comfeed Ltd.’s standard feed was ordered. Rats were often fed on broiler-II pellets (BR-II), which are made from a combination of corn, soybean meal, wheat germ, coconut meal, fish meal, meat meal, rice flour, tapioca, and premixes of coconut oil and fish oil.

#### 2.1.3. Instruments and Materials

Equipment used for phytochemical analysis and examination of the active substance content of CXBCH preparations included analytical balance (Ohaus Indonesia, Jakarta, Indonesia, type: EX224/AD), blender (Cosmos Indonesia, Jakarta, Indonesia, type:CB-812 G), 250 mL measuring cup (Iwaki Pyrex, Iwaki glas Indonesia, Jakarta, Indonesia), 100 mL glass beaker (Iwaki Pyrex, Iwaki glas Indonesia, Jakarta, Indonesia), electric stirrer (K-Ika), glass stirrer, Buchner funnel (Sigma-Aldrich, Merck-Indonesia, Jakarta, Indonesia), compressor, porcelain cup, fridge, filter paper, glass jar, and water bath, macerator pot, steam distillation set, Spectrophotometer UV-Vis (Hitachi high-tech Indonesia, Jakarta, Indonesia), Spectra Max M5 microplate reader (Molecular Devices LLC, San Jose, California, United States), GCMS (Shimadzu, type:QP201SE, Shimadzu (Asia pacific, Singapore), and TLC (Merck Indonesia, Jakarta, Indonesia). The quantities of thymoquinone, curcumin, polyphenols, and flavonoids in CX extract (CXE), BC extract (BCE), and CXBCH preparations were determined using thymoquinone (Sigma; Jakarta, Indonesia, cat. 274666-5G), curcumin (Sigma-Aldrich, Jakarta, Indonesia, cat:C1386), gallic acid (Sigma-Aldrich, Jakarta, Indonesia, cat: 398225), and rutin (Sigma-Aldrich, Jakarta, Indonesia, cat: R 5143) standards.

Equipment and materials for the chemopreventive test included rat cages containing husks, feed and drinking bowls, corn oil, CXBCH preparations, DMBA (Sigma-Aldrich, Jakarta, Indonesia, cat. D3254), distilled water, and nasogastric (NG) tube for DMBA treatment and induction. We also used a set of surgical instruments for organ harvesting, gloves, sterile disposable syringes, microspuit injector, glassware, mixer, vacutainer with anticoagulant, microtome, microscope, equipment for narcotics, ether or chloroform, 10% formalin as an organ fixation solution, and Hematoxylin and Eosin as coloring dyes for histopathological tests.

Materials and tools for antioxidant enzyme testing included Glutathione s—transferase assay kit (Cayman chemical company, Ann Arbor, Michigan, USA, catalog 703302); GST assay buffer (cat. 703310), GST sample buffer (cat. 703312), GST assay (control) (cat. 703314), GST glutathione (cat. 703316), GST CDNB (1-Chloro-2-4-dinitrobenzene) (cat. 703318), 96-well plate (colorimetric assay) (cat. 400014), 96-well cover sheet (cat. 400012); phosphate buffer (K3PO4); PBS (phosphate buffered saline); RPMI (Merck-Indonesia, Jakarta, Indonesia); centrifuge (Sorvall Biofuge Primo R, Marshall scientific LLC, Hampton, NH 03842, United States) micro pipette, and glassware. Griess A and B solutions for measuring NO levels were also used.

Instruments and materials for testing the immune response included a centrifuge (Sorvall Biofuge primo R, Marshall scientific LLC, Hampton, NH 03842, United States), 5–1000 µL micropipette with disposable tips, beakers, flasks, disposable tips, 5–10 mL pipettes, glassware, and plates with 24 wells and 96 wells. Solutions for cell culture included Tris Buffered Ammonium chloride (TBAH) and FBS (Fetal bovine serum) (Biochrom AG, Berlin, Germany); L-glutamine 200 mM (100×) (Invitrogen Europe Limited, Paisley, UK); amino acids solution (50×) (Invitrogen, Paisley, UK); Penicillin-streptomycin-solution (Invitrogen Europe Limited, Paisley, UK); Trypsin-EDTA (1×) (Invitrogen Europe Limited, Paisley, UK); Corn oil/corn oil; distilled water; sodium nitrate; trypan blue; PBS (phosphate buffered saline), and DMEM and RPMI growth media. Flow cytometry required the “Sorvall Biofuge primo R” centrifuge, 5–1000 L micropipettes with disposable tips, beakers, flasks, disposable tips, 5–10 mL pipettes, glassware, 15 and 50 L falcon tubes, vortex mixer and CO2 incubator, flowcytometer; freezer; eBioscience^®^ Flow Cytometry Staining Buffer (eBioscience, Thermo Scientific, Jakarta, Indonesia, Cat. No. 00-4222), and tritest reagent. 

### 2.2. Preparation of CXBC Herbal Honey, Phytochemical Analysis, and Active Substance Content Testing

The CXBCH preparations were formulated from a mixture of CX extract, BC extract, and honey and prepared by CV Al Afiat, a small traditional medicine business firm certified by the Food and Drug Supervisory Agency (FDSA) of the Republic of Indonesia. CXE and BCE were combined with honey solvent to create CXBC herbal honey. We used a mixture as an emulsifier to help the CX extract blend with the BC in the honey. The process of creating honey is summarized as follows: When we first prepared honey as a solvent, we weighed it according to our needs before adding tragacanth up to 5–10% of the weight of the honey as an emulsifier. Tragacanth was previously dissolved in water, which was then gradually added to the honey while stirring. All the ingredients, which have been weighed in accordance with the formula, were then gradually added to the honey, which was being swirled with a blower, after the tragacanth had been incorporated into it. Constant stirring was done to ensure that all components were thoroughly combined while maintaining a temperature of 30 to 40 °C.

As other researchers have previously done, phytochemical analysis was performed on CX extract, BC extract, and CXBCH preparations to determine the qualitative content of alkaloids, polyphenols, flavonoids, saponins, and triterpenes. Thin layer chromatography was used to perform preliminary phytochemical investigation on CXE, BCE, and CXBCH preparations. TLC was performed on aluminum-backed silica gel plates (Merck, Darmstadt, Germany, Art. 5533). The plates were heated at 103 °C for 3–4 min before being exposed to UV light (254 nm) or being sprayed with anisaldehyde-sulfuric acid reagent (anisaldehyde 0.5 mL, glacial acetic acid 10 mL, methanol 85 mL, conc. sulfuric acid 4.5 mL). As indicated by earlier researchers, we also used GC-MS and LC-HRMS analysis to identify the bioactive compounds in the CXBCH [49].

Folin–Ciocalteau reagent (Merck, Germany) and standard gallic acid (Sigma-Aldrich, cat:398225) were used to check the total phenol content. The calibration curve was prepared by mixing 90 μL of Folin–Ciocalteau reagent and 90 μL of NaCO3 solution with gallic acid. Ten milligrams of the sample were weighed, dissolved in 10 mL of ethanol, and homogenized. After filtering the material, the filtrate was used for analysis. Five hundred microliters of filtrate, 7.5 mL of distilled water, and 500 μL of Folin-Ciocalteu reagent were to be pipetted. After being homogenized and incubated for 8 min, the samples were analyzed. After incubation, 1.5 mL of sodium carbonate solution with a 20% concentration was added. After another incubation for 1 h, the absorbance was measured at a wavelength of 765 nm, and the concentration of polyphenols in the sample was calculated [50].

Rutin standards were used to measure the total flavonoid content using the Aluminum Chloride Colorimetric technique. Rutin (mg) equivalent was used to represent the total flavonoid concentration per gram of sample (1000 ppm). We made a 5% concentration stock sample solution (50,000 ppm). A 10 mL measuring flask containing 1 mL of the stock sample solution was pipetted into, filled with distilled water to the mark, and homogenized to create a sample solution with a 5000 ppm concentration. Before vortexing, 500 µL of the 5000 ppm sample solution and 100 µL of the 10% AlCl3 reagent solution were pipetted. The preparation was then mixed with 100 µL of 1 M sodium acetate reagent, vortexed, and finally, 2.8 mL of distilled water was added before the absorbance was calculated [51].

The thymoquinone levels were measured with UV-vis spectrophotometry with the same procedure as carried out by previous researchers. As much as 12.5 mg of thymoquinone was weighted and then dissolved in methanol to a final volume of 25 mL to obtain a concentration of 500 µg/mL. The thymoquinone level calibration curve was generated by diluting the 500 µg/mL concentration into five different thymoquinone concentrations. A 0.1 mL thymoquinone mother liquor was pipetted into a 25 mL volumetric flask, dissolved in methanol to a final volume of 25 mL, and then quantified using a UV-Visible spectrophotometer at a wavelength of 200–400 nm to find out the specific wave length number of thymoquinone. The thymoquinone levels in CX extract, BC extract, and CXBCH preparations were carried out as follows: the sample was prepared by weighing a certain amount of BC extract and then dissolved in methanol to a final volume of 10 mL. The test material was then homogenized with a vortex for 2 min. After being allowed to stand for 1 min, the methanol layer at the top was taken out. After that, the sample was filtered using a 0.45 m syringe filter and then injected into High-Performance Liquid Chromatography (HPLC) with an injection volume of 20 µL, and the peak area was seen. The peak area obtained was then substituted into the regression equation on the calibration curve as the Y value to obtain the sample concentration in ppm. The % *w*/*w* concentration was calculated [52].

The chromatographic method was used to determine the curcumin levels in CXBCH preparations. We started the process by determining the wavelength and calibration curve by analyzing the standard solution with a UV-Vis chromatography instrument three times. The average results of the measurements were used as a reference in the analysis of curcumin with HPLC. Calibration curves were made using a series of reference standard solutions with five different concentrations (0.5, 1, 2, 5, and 10 µg/mL). HPLC conditions were column E-C18, column temperature of 40 °C, mobile phase mixture of acetonitrile and acetic acid 2% (55:45), and flow rate of 0.5 mL/minute with a UV-Vis detector at a specified wavelength. Curcumin content was determined by entering the average value of the sample area from three replications into the linear regression equation from the standard curve so that the content was obtained in g/mL units. The results obtained were then converted into ppm units. Each prepared sample was put in ultrasonic degassing to remove air bubbles. The sample was then filtered with a 0.45 m syringe filter, injected into the HPLC system at 10.0 µg/mL, and replicated three times [53]. 

At the National Research and Innovation Agency, Yogyakarta, Indonesia, the LC-HRMS analysis of ethyl acetate and the aqueous fraction was carried out using a Thermo Scientific™ Acclaim™ VANQUISH™ PM 100 C183 µm × 150 mm Q Exactive Orbitrap HRMS. Gas temperature: 30 °C, gas flow: 11.01/min, nebulizer: 40 psi, VCap: 3500, fragmentor: 175, skimmer 1: 65.0, and octupole RF Peak: 750 were the settings for the source and scan parameters. Acetonitrile, a 5 mM acetate buffer, and water were used to elute the solvent at a flow rate of 1.5 mL/min. Starting with 5% acetonitrile for 0.1 min, the elution gradient was increased to 30% acetonitrile for 10 min, 80% acetonitrile for 32 min, and then returned to its initial settings. The column temperature was maintained at 30 °C throughout the entire procedure. The flow cell of the diode array detector was traversed before the column elution was sent to a Q-TOF HRMS equipped with an electrospray interface. With a scan rate of 1.03 and a mass range of 100–2000 Daltons, positive electron spray ionization (ESI-positive mode) was used to analyze the mass spectrum [54].

### 2.3. Reactive Radical Binding Activity Test of CXBCH Preparations

CX extract (CXE), BC extract (BCE), and CXBCH preparations were tested for reactive radical binding activity. Samples were made of 100 ppm mother liquor by dissolving 10 mg of extract in 100 mL of methanol PA. Furthermore, dilution using a methanol pa solvent was achieved with varying concentrations of 5 ppm, 6 ppm, 7 ppm, 8 ppm, and 9 ppm. DPPH stock solution was prepared by dissolving 5 mg of solid DPPH into 100 mL of methanol PA. Then, a comparison solution was prepared: a control solution containing 2 mL of methanol PA and 1 mL of 50 ppm DPPH solution. Every 2 mL of sample solution and 2 mL of DPPH solution were prepared for the test sample. Then, it was incubated for 30 min at 27 °C until there was a color change from DPPH activity. All the samples were made in triplicate. Samples of the extracts and CXBCH preparations that had been incubated were then tested for absorbance values using a UV-vis spectrophotometer at a wavelength of 517 nm. The IC_50_ value is the sample concentration required to scavenge 50% of DPPH free radicals, which we calculated by plotting the percent inhibition against the log sample extract concentration [55].

### 2.4. Viability and Chemopreventive Mechanism Test of CXBCH

Cytotoxicity activity of CXBCH preparation was tested out on T47D, Hela, and HTB-183 cells. Cancer cells were cultured in DMEM containing 10% FBS supplemented with 1% penicillin (100 units/mL) and streptomycin (100 µg/mL) at 37 °C, 5% CO_2_ in an incubator. The test material’s cytotoxicity activity was determined using the 3-(4,5-dimethyl-2-thiazolyl)-2,5-diphenyl-2-H-tetrazolium bromide (MTT) assay method. Briefly, T47D, Hela and HTB-183 cells were plated in 96-well plates (5 × 103 cells/well) in DMEM containing 10 % FBS and 24 h later they were treated with CXBCH (0, 125, 250, 500, 10,000 μg/mL) and incubated for 72 h in DMEM with 1% FBS. Then, the MTT reagent (0.2 mg/mL) was added to each well and incubated for 4 h. In addition, 200 µL of dimethyl sulfoxide (DMSO) reagent were added to dissolve the formazan product in each well, followed by measuring the absorbance at a wavelength of 595 nm using a spectrophotometer (SpectraMAX M5, Molecular Devices, CA, USA) [56]. 

On Hela cells, we also noticed CXBCH’s chemopreventive agent mechanism of action. Using flow cytometry, we evaluated the impact of administering CXBCH preparations at concentrations of 1/2 IC_50_ and IC_50_ on the expression of p53 and caspase-3 [57]. 

### 2.5. Chemopreventive Testing in Animal Models of Cancer

#### 2.5.1. DMBA Induction and CXBCH Administration in Experimental Animals

After undergoing quarantine for one week, a total of 80 Sprague Dawley (SD) rats aged four weeks were then randomly grouped into eight groups of ten rats. Group I was the normal group where female SD rats received standard food and drink. Group II was given a dose of CXBCH1 (equivalent to 1 × 5 mL/70kg BW), Group III a dose of CXBCH2 (equivalent to 2 × 5 mL/70kg BW), and Group IV a dose of CXBC3 (3 × 5 mL/70 kg BW) as the treatment groups. Because each set of test animals weighed 100 g, a 1 mL solution comprising 0.09 mL of CXBCH was administered along with 1/2 mL, 1 mL, and 1.5 mL of CXBCH1, CXBCH2, and CXBCH3, respectively. Group V, as the positive control group, received thymoquinone orally at 20 mg/kg BW [39]. Group VI, as the positive control group, was given tamoxifen 0.6 mg/kg BW/day [40]. CXBCH, thymoquinone and tamoxifen were administered for five weeks during DMBA induction followed by four weeks post-induction. As the negative control group, Group VII received DMBA 2 × 20 mg/kg BW/week for five weeks and standard food and drink [11]. Group VIII was the solvent control group, where the test animals received standard food, drink and solvent (corn oil). Each SD rat was given a maximum volume of 2 mL that contained the active ingredients according to the dose [34].

All groups, except the normal and solvent control groups, were induced with DMBA. Carcinogenesis experiments used the carcinogen DMBA 20 mg/kg BW, which was administered intragastrically with a probe twice a week for five weeks. Observations on tumor formation started from the last DMBA administration until the 30th week of treatment. 

#### 2.5.2. Examination Procedure of the Chemopreventive Effect of CXBCH Preparations

##### Observation of Clinical Manifestations and Nodule Formation

Examination of the main clinical manifestations was carried out on body weight, survival, and biochemical features for the physiology of the kidney, liver, and peripheral blood. Body weight measurements of each rat were carried out twice a week. Peripheral blood and blood chemistry examinations were carried out at the Integrated Testing and Examination Institute Unit I. Peripheral blood examinations were carried out using a Sysmex KX-21 hematology analyzer (Sysmex Inc., Sysmex Indonesia, Jakarta, Indonesia), while blood chemistry examinations (SGPT, SGOT, urea, and creatinine) using a spectrophotometric device (Microlab 300, Elitech-Indonesia, Jakarta, Indonesia). Blood samples were taken from the rat through the orbital sinus as much as ± 1.5 mL. Blood from the orbital vein was collected in a labeled Eppendorf tube containing an anticoagulant and then divided into two: one part for examination of peripheral blood images and another part for blood biochemical examination. The blood for blood biochemical examination was allowed to stand for 15 min, before being centrifuged at 4000 rpm for 10 min (1789 G), and then the supernatant (serum) was taken and used to determine SGPT, SGOT, urea, and creatinine levels [34,41].

##### Examination of Nodule Incidence and Multiplication

The antitumorigenic activity of the test materials was observed clinically, macroscopically, and microscopically. The macroscopic observation was carried out by palpating the mammary organs, measuring the formation of tumor nodules (incidence), and counting the number of nodules formed (nodule multiplicity) in the breast tissues. The day or date when the tumor nodule was first seen or felt and the number of tumor nodules were recorded accordingly. Observation of the tumor nodules was carried out after the DMBA administration was complete, starting from the eighth week of the experiment by observing and palpating. The presence of new nodules in the mammary organs was counted as the incidence of tumor nodules. The chemopreventive effect of the CXBCH preparations was expressed by (i). the incidence of nodule formation between the treatment groups and the DMBA group; (ii). the number of tumor nodules per group and tumor multiplicity; and the (iii). time of nodule formation [42,58].

##### Histopathological Examination

Histopathological examination was carried out at the 30th week of the experiment to determine changes in the structure of tissues and cells in the test animals’ mammaries. Organ harvesting was carried out as follows: rats were sacrificed, and their stomach skins were cut. The tumor nodules or mammary tissues that needed to be examined were removed, and then cleaned with 0.9% of NaCl solution, and put in a pot containing 10% formalin. Technicians made histopathological preparations at the Anatomical Pathology Laboratory, Faculty of Medicine, Universitas Gadjah Mada (UGM), Yogyakarta. The fixation process was carried out on the mammary organs. After the fixation process, trimming or thin cutting of tissue approximately 4 mm thick was carried out using a scalpel knife No. 22–24. The tissue was loaded in an embedding cassette which functioned as a network holder.

Tissue dehydration was carried out in a tissue processor after trimming using the dehydrating liquid, ethanol, to remove water contained in the tissue. This dehydrating liquid was then cleaned with a cleaning reagent, namely toluene, which would be replaced with paraffin by penetrating the tissue. This process is called impregnation. The tissue was put in hot paraffin, which would infiltrate the tissue. This process is intended to make it easier to cut the tissue using a microtome. The tissue was then cut using a microtome knife with a thickness of 5 µm. The layer was then placed on a slide to color. Staining was performed using Hematoxylin and Eosin. After the tissue on the slide was stained, mounting was carried out by dripping the mounting material and covering it with a cover glass. After the preparations have finished, tissue inspection and shooting were carried out at the Pathology Laboratory of the Faculty of Veterinary Medicine, Gadjah Mada University (UGM). Examination of the tissue using a light microscope was carried out by an Anatomical Pathologist from the Faculty of Veterinary Medicine, Gadjah Mada University, and the photography was also carried out at the same laboratory. Microscopic observations included the histological condition of the mammary gland organs from the H&E staining. H&E preparations were observed descriptively to determine the carcinogenesis stages. Upon microscopic observations based on the proliferative level of epithelial cells, the tissue was categorized as normal, hyperplasia/dysplasia, or adenocarcinoma according to the histopathological appearance [43,59]. 

### 2.6. CXBC Antioxidant Activity Examination Procedure

#### 2.6.1. Examination of Serum NO Levels

Examination of serum NO levels was carried out using the colorimetric method on the blood samples taken through the orbital vein. As much as two cc of blood was put into a blood collection tube, and a colorimetric determination of nitric oxide levels was carried out using Griess’s solution [44,60].

#### 2.6.2. Liver and Spleen GST Enzyme Activity

As previously investigated, GST enzyme activity was determined by enzymatic analysis [45]. SD mice that had been treated for seven weeks and induced with DMBA at the end of the 30th week, the day before data collection, fasted for 24 h. Then, the test animals were decapitated, their liver and spleen tissues were removed, and samples were made. As much as 1 g of liver or spleen tissue was taken from the cytosolic fraction of the liver microsomal to measure the total GST enzyme activity. Then, the samples were washed with PBS. After being considered clean, the tissue was homogenized in 5–10 mL of cold buffer (100 mM K3PO4, pH 7.0, containing two mM EDTA) and centrifuged at 10,000× *g* for 15 min at 4 °C. The liver homogenate supernatant obtained was then examined for the GST enzyme activity using the GST ELISA kit following the industry standard procedure. The speed of GST enzyme activity was determined based on the formation of GSH conjugation with 1-chloro-2-4-dinitrobenzene (CDNB). The final assay volume was set at 200 μL per well.

The room temperature for the test was 25 °C. GST activity check steps were carried out according to the standard instructions from the industry. Each well was filled with 150 µL of assay buffer, 20 µL of glutathione, and 20 µL of the sample. The reaction was initiated by quickly adding 10 µLCDNB to each well. Then, the microplate was shaken for a few seconds to corrode the test material. The reaction results were read every minute (at least 5×) with an Elisa reader at a wavelength of 340 nm. The GST reaction rate on an ELISA reader at a wavelength of 340 nm can be determined using the CDNB extinction coefficient of 0.00503µM-1. At 25 °C every minute, 1 unit of enzyme will conjugate with 1 nmol of CDNB by reducing glutathione. GST activity was calculated using the following formula:

GST activity = ΔA340/min × 0.2 mL × sample dilution

0.00503 µM^−1^ × 0.02 mL

### 2.7. Monitoring the Immune Response

#### Number and Types of CD4, CD8 and CD4CD25 Lymphocytes by Flowcytometer

Examination of the number and types of leukocytes was carried out using a Sysmex KX-12 hematology analyzer in the Integrated Research and Testing Laboratory (IRTL), Gadjah Mada University. We examined the number of CD4, CD8, CD4CD25, and CD8CD25 by flow cytometry in the Clinical Pathology Laboratory, Gadjah Mada University [46,61].

Blood that had been collected in a vacutainer tube containing an anticoagulant was then examined with a flow cytometer with the following procedure: (i). As much as 50 µL of the test material/specimen was pipetted into a falcon tube; (ii). A total of 10 pL of the CD4/CD8 FITC/CD25 tritest reagent per CP was added to each falcon tube that had been filled with the test material; (iii). The specimens and tritest reagents in the Falcon tube were mixed until homogeneous with a vortex mixer, then incubated for 15 min at 20–25°C and dark room; (iv). While waiting for incubation, the FACS reagent was diluted, where 50 pL of FACS solution were diluted 10× by adding 450 pL of distilled water, then mixed until homogeneous; (v). After the incubation time was complete, the sample was added with 450 µL of the already diluted FACS reagent (lx); (vi). After adding the FACS reagent to each falcon tube, the sample was mixed until homogeneous with a vortex mixer, then incubated for 15 min, at a temperature of 20–25 °C, in a dark room; (vii). After the incubation was over, analysis was performed using BD Biosciences FACS and CellQuest software to determine CD4/CD8/CD25 counts.

### 2.8. Data Analysis

The bioactive content of TLC results and measurements of total flavonoids, polyphenols, curcumin and thymoquinone are presented descriptively. Raw data files acquired from the LC-HRMS were processed using MZmine 2 and then Mestre Nova 12.0 for compound annotation using Dictionary of Natural Products 2, ChemSpider, and METLIN database. 

Using one-way ANOVA, the test findings of cell viability and antioxidant capacity were compared between groups for various means. After establishing the linear regression line equation for the correlation between the percentage of viability and the log concentration of CXBCH preparations, we also determined the IC_50_. The CXBCH preparation’s antioxidant activity was also tested using the same calculation.

The nodule number and weight were then given in a descriptive manner after the tumor incidence was expressed as a percentage of the tumor occurrence in each group. By characterizing cell proliferation activity, metaplasia, mutations or neoplasms, and neoplasms progress, the results of histopathological observations of tumor nodules were assessed descriptively and qualitatively. If there was no change in the proliferation, it was expressed as normal proliferation, and if there was an increase in activity, it was expressed as hyperproliferation.

The animal laboratory data (hemogram profile, blood chemistry, NO level, GST level, number of CD4, CD8, and CD4CD25 cells) were tested for normality and homogeneity using the Kolmogorov–Smirnov Test and the Levene’s Test, respectively. If the data were normally distributed and homogeneous, a parametric test with a One-Way Analysis of Variance (ANOVA) was used to perform the statistical analysis between dosage groups. If the data were not homogeneous or normally distributed, a non-parametric test with a Kruskal–Wallis’s test was performed to examine the difference between the dosage groups. Finally, the Mann–Whitney post hoc test was used to determine the mean difference between the two groups.

The body weight data of the test animals were normally distributed, while the hemogram profile, blood chemistry (urea, creatinine, SGOT, SGPT), CD4 cells, CD8 cells, CD4CD25 cells, nitric oxide levels, and glutathione s transferase levels were mostly abnormally distributed. We attempted to transform laboratory data and hemogram profiles, but the transformed data remained irregularly distributed. The repeated measure method was used to assess data on body weight development. The mean differences between groups were examined with Kruskal–Wallis for the blood cell count, SGPT, SGOT, serum urea, creatinine, GST level, NO levels and CD4, CD8, and CD4CD25. A 95% confidence level was used for all statistical tests.

## 3. Results

### 3.1. Active Substance Content of CXBCH Preparations

The active substance contents of CXBCH preparations were tested qualitatively and quantitatively.

Thin layer chromatography was used to qualitatively assess the active ingredient of CXBCH, CXE and BCE. Supplementary Table S1 shows the findings of the qualitative and quantitative analysis of the active substance content. 

The results of the phytochemical analysis (Supplementary Table S1) show that the CXBCH preparations qualitatively contained alkaloids, flavonoids, phenolics, saponins, and triterpenoids. It is known quantitatively that the CXBCH preparation contains 38.87 ppm of polyphenols, 56.86 ppm of flavonoids, 46.45 mg/mL of thymoquinone, and 68.86 mg/mL of curcumin.

#### 3.1.1. Bioactive Compound Profile on CXBC with GCMS and LC-HRMS

We have observed the volatile compound in the CXBCH preparation using GCMS. The figure and table show the findings of the profile of the volatile compound on CXBCH (Figure S1).

Observations using the GCMS tool obtained data for more than 30 volatile compounds from CXBCH preparations. The major compounds present in the CXBCH were 9-Hexadecenoic acid (33.65%), Hexadecenoic acid (16.49%), Ethyl linoleate (10.99%), octadecanoic acid (8.88%), gamma-curcumene (6.82%), benzene, 1-(1,5-dimethyl-4-hexenyl)-4-methyl (4.14%), and Hexadecenoic acid, ethyl ester (3.36%). The entire list of compounds observed with GCMS is presented in Supplementary Table S2.

Based on the literature search, some compounds found in the CXBXH have been reported to display antioxidant, chemopreventive, anticancer, anti-inflammatory, immunomodulatory, antibacterial, and antimicrobial activities. For example, *n*-hexadecenoic acid, gamma-curcumene, methyl ester eicosadienoic acid, Cyclopropaneoctanoic acid and ethyl-cyclodocosane were some of the compounds identified by GCMS (Table S3) and in CXBCH preparation. Anti-inflammatory as well as anti-cancerous compounds identified included, 9,12-octadecadienoic acid (z,z), octadecanoic acid, heptadecyl trifluoroacetate and alloaromadendrene. Major compounds of CXBCH were revealed to be 6.12 9-Hexadecenoic acid (33.65%), 4.41 Hexadecenoic acid (16.49%), and 3.13 Ethyl linoleate (10.99%).

#### 3.1.2. Profile of Bioactive Compounds in CXBCH Preparations Observed Using Liquid Chromatography High Resolution Mass Spectrometry (LC-HRMS)

The profile of bioactive compounds in CXBCH preparations observed with LC-HRMS is presented in Figure S2 and Table S3.

Formula, molecule name, RT, annotation delta mass and max area (absolute) observed with LC-HRMS from CXBCH preparations are presented in Table S3. According to Table S3, more than a hundred active chemicals can be found when the active compounds in CXBCH preparations are examined using LC-HRMS. Linoleic acid, eremantin, anhydro-D-fructose, 1,5-Anhydro-6-deoxy-D-threo-hex-1-en-3-ulose, 1-linoyl glycerol, monoolein, Turmerone, meglutol, and L-palmitin are the 10 most abundant active components in CXBCH. According to the results of the LC-HRMS analysis of the CXBCH preparations, the three primary components of CXE are turmerone (order 8), curcumin (order 13), and curcumene (order 15). Quercetin, the primary flavonoid, is ranked 105th, and thymoquinone, the primary active ingredient in BCE, is ranked 137th.

### 3.2. Cytotoxic and Antioxidant Activity of CXBC Preparations

The antioxidant activity of CXBCH preparations was tested using the DPPH method while the cytotoxic activity test was carried out on T47D and Hela cells.

#### 3.2.1. Radical Scavenging Activities CXBCH Preparation

**Figure 1:**
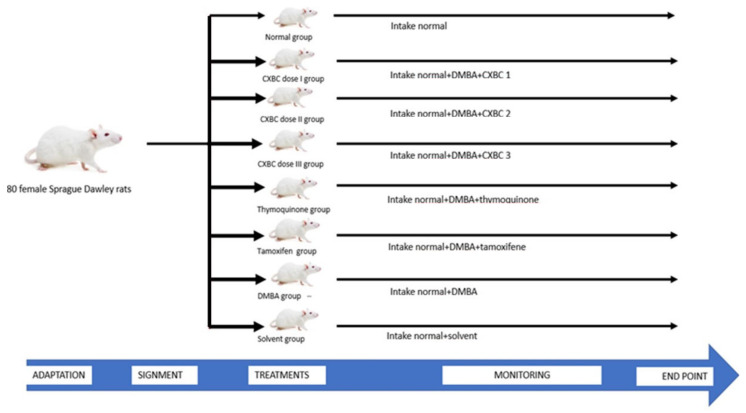
Placement and treatment of test animals.

Figure 1 shows the ability of CXBCH, CXE, and BCE preparations as scavengers of free radicals from DPPH.

**Figure 2:**
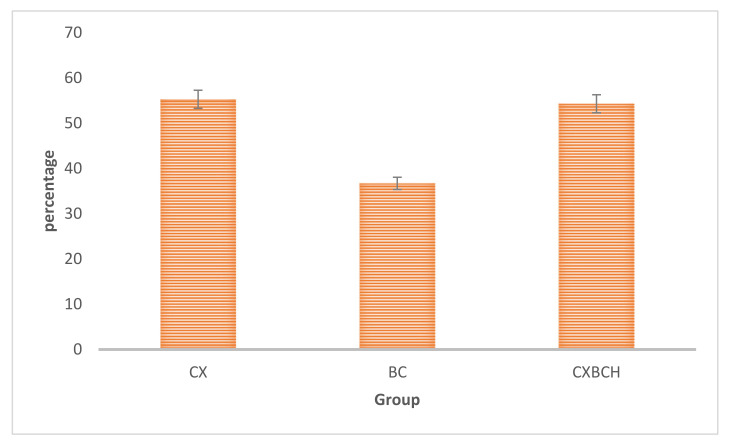
Antioxidant activity (IC50, mcg/mL) of CX extract, BC extract and CXBCH preparation using the DPPH method.

Based on Figure 2, it can be seen that the ability (IC_50_) of the CXBCH preparation to bind free radicals was 54.26 mcg/mL.

#### 3.2.2. Cytotoxicity Activity of CXBCH Preparation

**Figure 3:**
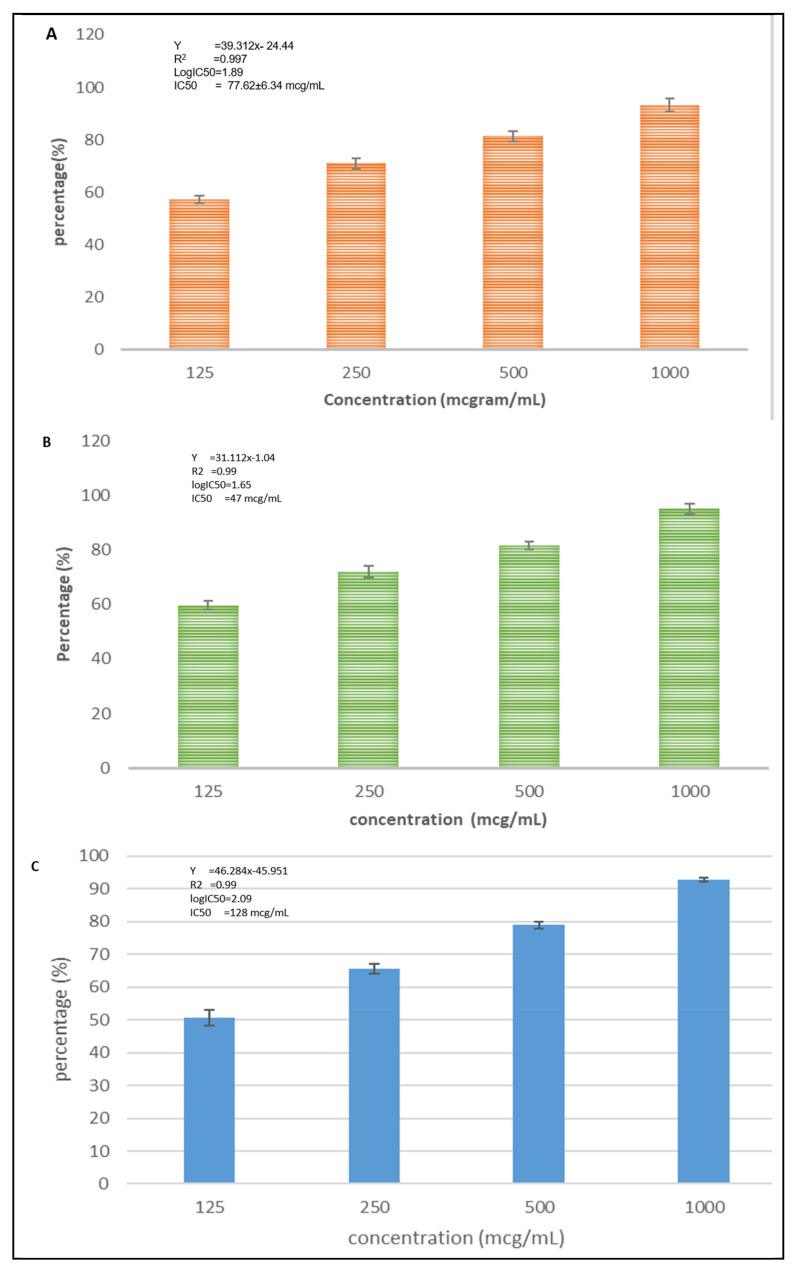
Inhibition activity of CXBCH preparation on T47D (**A**), Hela (**B**), and HTB-183 (**C**). The findings of the one-way mean difference test between concentrations According to an ANOVA analysis, the three cell types were tested with a *p*-value < 0.05.

Figure 3 shows that the CXBCH preparations inhibited the growth of T47D, Hela, HTB-183 cells.

Based on Figure 3, we know that the CXBCH preparations inhibited the growth of T47D, Hela, HTB-183 cells with an IC_50_ of 77.62 ± 4.66, 47.34 ± 13.29, and 128 ± 12.52 mcg/mL, respectively.

**Figure 4:**
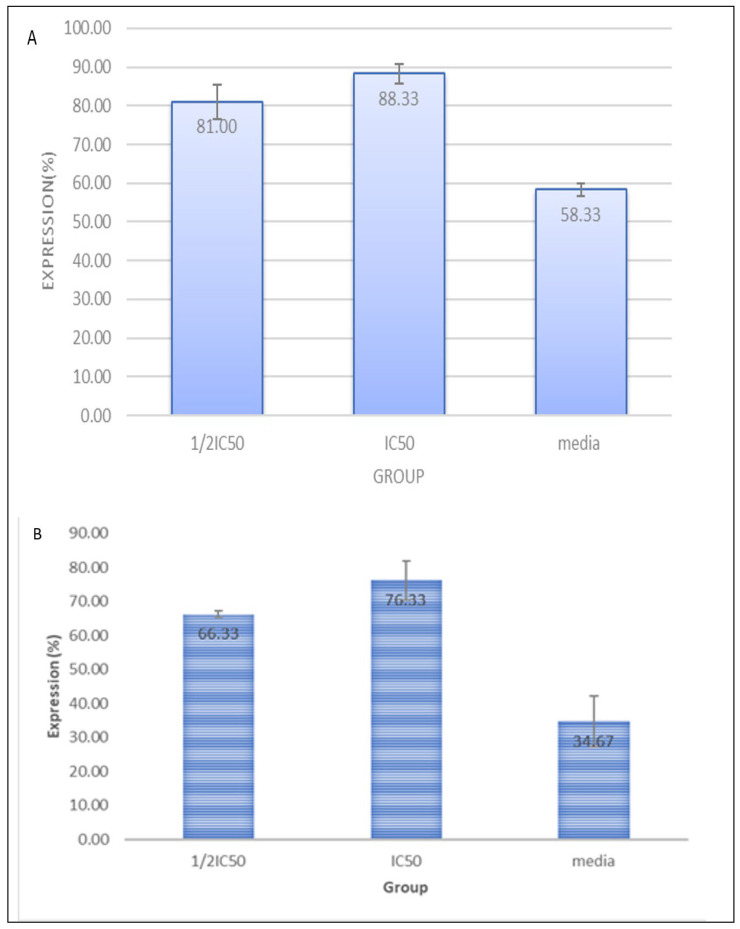
Expression of p53 (**A**) and Caspase-3 (**B**) in Hela cells exposed to CXBCH preparations.

Furthermore, we conducted an in vitro test to determine the mechanism of the chemopreventive action of CXBCH preparations by observing immunocytochemistry of p53 and caspase-3 expression on Hela cells. The results of testing the effect of CXBCH preparations on p53 expression in Hela cells are presented in Figure 4.

Caspase-3 is a crucial protein in apoptosis in addition to p53. Important mediators of programmed cell death are caspases. Among these, caspase-3 is a death protease that is regularly activated and catalyzes the precise cleavage of numerous essential cellular proteins. The results of the experiment demonstrated that the CXBCH preparations boosted the expression of caspase-3 and p53.

### 3.3. CXBCH Chemopreventive Activity in SD Rats

#### 3.3.1. Clinical Conditions of Test Animals

**Figure 5:**
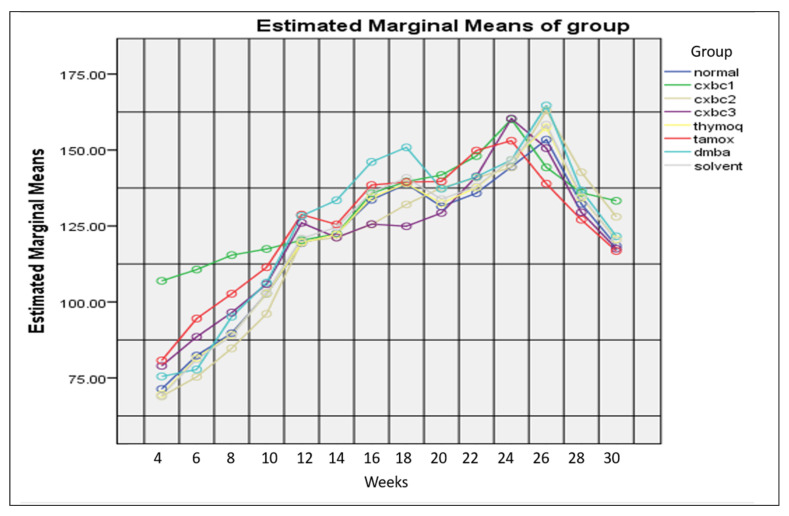
Development of DMBA-induced SD rat body weight by administering CXBCH preparations.

Figure 5 depicts the weight gain of the test animals over 30 weeks of monitoring. Based on measurement in the first week, the average body weight of the SD rats was virtually similar between groups (*p* > 0.05). In general, the average body weight from the first week to the 26th week increased but from the 26th to the 30th week it decreased. The average body weight in the DMBA and tamoxifen groups from the 26th to the 30th week was the lowest, but not statistically significant (*p* > 0.05). 

Table 1 presents the survival ability of DMBA-induced female SD rats with CXBCH treatment.

Table 1 illustrates that the DMBA group had the lowest survival rate (0%) followed by the tamoxifen group with three deaths (70%), and the CXBCH2 group with two deaths (80%), while the CXBCH3 group had the highest (*p* < 0.05 The CXBCH1, the normal, the solvent control, and the thymoquinone groups all had the highest livability rate (90%), with only one death in each group. These results indicate that DMBA induction increased the risk of death and the CXBCH administration increased the survival rate. 

The examination results of peripheral blood, kidney and liver function of SD rats are presented in Table 2 and Table 3. DMBA induction at 10 × 20mg/kg BW, 2×/week for five weeks in female SD rats, reduced hemoglobin (Hb), mean corpuscular volume (MCV), mean corpuscular (MCH), and blood cells, but increased the levels of SGPT/SGOT and urea/creatinine. This study proved that DMBA induction suppressed bone marrow hematopoiesis or hematotoxicity. The number of leukocytes, erythrocytes, platelets, Hb, MCV, and MCH in the DMBA group was lower than that in the normal group (*p* < 0.05). The administration of CXBCH, thymoquinone, and tamoxifen for two weeks before and five weeks during DMBA induction increased cell count, Hb, MCV, and MCH, as the average leukocyte, erythrocyte, platelet, Hb, MCV, and MCH counts of the treatment groups were higher than that of the DMBA group (*p* < 0.05).

The results of renal and hepatic physiology examination are presented in Table 3, showing that DMBA induction is nephrotoxic and hepatotoxic. However, the serum urea and creatinine levels in this study differ from the referenced study. DMBA induction increases serum urea and creatinine levels, as shown by the significantly higher levels of urea and creatinine levels in the DMBA group compared to the normal group (*p* < 0.05). Furthermore, the average levels of SGPT and SGOT in the DMBA group were also 3× and 7× higher respectively than those in the normal group (*p* < 0.05).

Administration of CXBCH for two weeks before and five weeks during DMBA induction in SD rats was shown to be nephroprotective and hepatoprotective. The average urea and creatinine levels in the CXBCH groups were lower than those in the DMBA group (*p* < 0.05). The mean blood urea and creatinine levels in the thymoquinone group did not differ from those in the CXBCH groups (*p* > 0.05) but those in the tamoxifen group were higher (*p* < 0.05). The average levels of SGPT and SGOT in the CXBCH groups were significantly lower than those in the DMBA group (*p* < 0.05). It appears that administration of CXBCH has decreased SGPT and SGOT levels in DMBA-induced SD rats, 66% and 90% respectively.

#### 3.3.2. Nodule Formation

The examination results of the percentage of nodule formation, the number of nodules per group, and the nodule weight are presented in Table 4 and Supplementary Figure S3.

The DMBA group had the highest percentage of nodule formation, with 100%. All the SD rats in the DMBA group were successfully induced with DMBA and all formed tumor nodules (100%). The administration of CXBCH to DMBA-induced female SD rats reduced the percentage of nodule formation per group, as shown by the lower percentage of nodule formation in the CXBCH treatment groups. Furthermore, the thymoquinone and tamoxifen groups had the lowest nodule formation percentages, with 28% and 30%, respectively. Therefore, the CXBCH administration for two weeks before and five weeks during DMBA induction inhibited the formation of tumor nodules in SD rats.

Based on the number of nodules formed per group, the DMBA group had the highest number, with 14 nodules. The number of nodules in the CXBCH1 (8 nodules), CXBCH2 (8 nodules), CXBCH3 (6 nodules), thymoquinone (3 nodules), and tamoxifen (5 nodules) groups was lower than that in the DMBA group. Among the groups that received CXBCH, CXBCH3 had the least number, with six nodules. The results of this study indicate that DMBA can induce the formation of tumor nodules in the mammaries of SD rats, similar to what has been reported by previous researchers.

Based on the time of tumor nodule formation, the earliest nodule formation occurred in the DMBA group, namely at the 10th week, followed by the CXBXH1 group at the 14th week, the CXBCH3 group (17th week), CXBCH2 (18th weeks) and tamoxifen (18th week). The thymoquinone group had the most recent formation of tumor nodules, namely after the 20th week. Among the treatment groups that received CXBCH, nodule formation was formed the fastest in the CXBCH1, followed by the CXBCH3 and CXBCH2. 

#### 3.3.3. Histopathological Examination of Tumor Tissue in SD Rats Induced with DMBA

**Figure 6:**
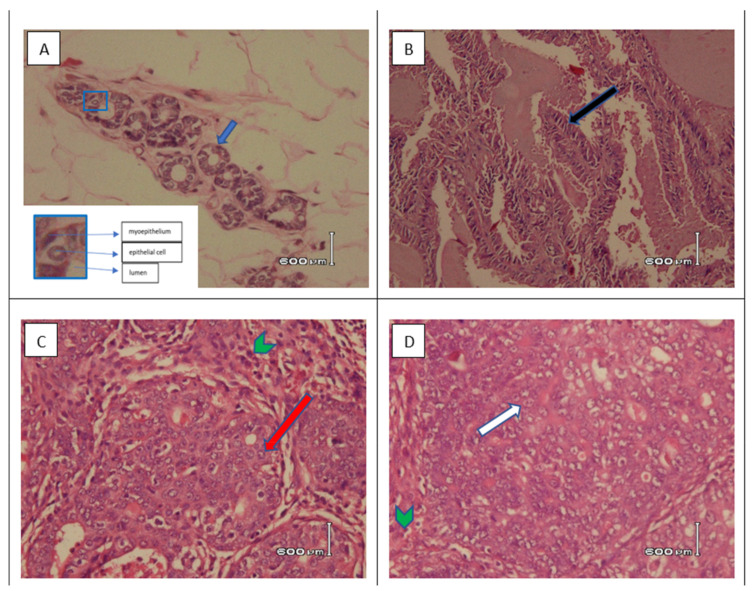
Microscopic view of mammary tissue of SD rats induced by DMBA by giving CXBC for two weeks and five weeks during induction (HE staining). Notes: (**A**) normal tissue (400×). Mammary gland ducts are composed of non-atypia epithelial cells and myoepithelium (insert), (**B**) tissue with papillary carcinoma (100×). Delicate papillary fronds (black arrow) and expansile papillary tumor, with low or intermediate grade nuclei, cuboidal to columnar epithelial cells that lacks myoepithelial cells along the papillae and at the periphery or shows focal peripheral myoepithelial staining; (**C**) tissue with Ductal carcinoma in situ (400×). An intraductal epithelial proliferation with intermediate grade nuclear atypia (red arrow), tubular and cribriform growth pattern with sufficient lymphocyte reaction (green arrow head); (**D**) tissue with invasive ductal carcinoma of no special type (NST) (400×). The preparation shows solid and tubular epithelial tumor tissue, infiltrative to the surrounding connective tissue. Tumor cells are atypia, polymorphic, large size. Cytoplasm a little until enough. The nuclei (white arrow) are large, pleomorphic, round, oval, polygonal, irregular chromatin, partly vesicular with nucleolus are clearly visible. Mitosis is slight. Lymphocyte reaction is slight (green arrow head).

Figure 6 and Table 5 present the histopathological observations of carcinogenesis in mammary tissue tumor nodules. None of the SD rats were diagnosed with mammary carcinoma (adenocarcinoma) in the solvent and normal groups as microscopically, the mammary gland cells of SD rats in these groups showed normal mammary tissue histology. There was no change in the histology of mammary tissue characterized by hyperplasia, metaplasia, and neoplasms. Meanwhile, the mammary tissue of SD rats in the DMBA group, which experienced carcinogenesis and formed tumor tissue, showed hyperplasia of the connective tissue. The connective tissue was denser due to pressure from the enlarged tumor cells. Neoplasm cells from the ductal epithelium (adenocarcinoma) and acini were seen as well as the presence of inflammatory cells and collections of necrotic cells (Figure 6).

The study found that seven SD rats in the DMBA group ultimately all had nodules (100%), which were 100% histopathologically diagnosed as adenocarcinoma. Administration of CXBCH for two weeks before and five weeks during DMBA induction could inhibit the carcinogenesis process (Figure 6). Histopathological examination of mammary tissues in the treatment groups with various CXBCH doses showed tumors with adenocarcinoma, tumors without adenocarcinoma, and those that showed hyperproliferation and normal.

Histopathological examination of the mammary tissues in the CXBCH treatment groups revealed that there were various forms of epithelial proliferation, both epithelial from the acini and ductal epithelium. However, some of the hyperplastic features seen in the thymoquinone, tamoxifen, CXBCH1, CXBCH2, and CXBCH3 groups could not be classified as mammary adenocarcinoma. Histopathological picture of the hyperplastic epithelium was found in the treatment groups that received CXBCH1 (30%), CXBCH3 group (40%), and CXBCH2 (10%). In the thymoquinone group, the hyperplastic picture was 30%. Hyperplasia was not found in the tamoxifen and DMBA groups. In the mammary gland tissues, ductal adenocarcinoma in situ and invasive was found. As observed in the CXBCH and tamoxifen groups, anaplastic cells were still restricted to the lobules and the ductal basement membrane remained intact in adenocarcinoma in situ. The percentage of adenocarcinoma formation among SD rats that received successive CXBCH treatment was the lowest in the CXBCH3 group at 10%, the CXBCH1 group at 20%, and the CXBCH3 group as the highest at 30%. The DMBA group exhibited the highest percentage of invasive cancer on histopathology (100%) compared to the thymoquinone group (0%). This study demonstrated that CXBCH injection slowed the progression of carcinogenesis in DMBA-induced SD rats, indicating that CXBCH could potentially serve as a chemopreventive drug.

### 3.4. CXBCH by Increasing GST Activity and Decreasing Serum NO Levels

The results of an examination of serum NO levels of SD rats at the 30th week of treatment are presented in Table 6. The results indicate that CXBCH1, CXBCH2, and CXBCH3 administration for seven weeks to female DMBA-induced SD rats decreased serum NO levels.

DMBA induction at 2 × 20 and 10 × 20 mg/kg BW in SD rats increased serum NO levels. The results of this study proved that the serum NO levels in the DMBA group at the fourth week of measurement were higher than those in the normal and solvent control groups (*p* < 0.05). Likewise, at the 30th week of measurement, the serum NO level of the DMBA group was higher than that of the normal and solvent control groups (*p* < 0.05). Administration of CXBCH, thymoquinone, and tamoxifen was shown to reduce serum NO levels of DMBA-induced SD rats. At the 30th weeks of measurement, the serum NO level of the treatment group receiving CXBCH, thymoquinone, and tamoxifen was lower than that of the DMBA group (*p* < 0.05).

CXBCH preparations increased the activity of GST enzymes in the liver and spleen, as presented in Table 7. In general, this study proved that the liver GST enzyme activity in the DMBA group was lower than the regular liver GST activity (*p* < 0.05). In other words, DMBA induction decreases hepatic GST enzyme activity. 

CXBCH administration increased the activity of the liver GST enzyme in SD rats, as the average liver GST enzyme activity of the CXBCH groups was higher than that of the DMBA group (*p* < 0.05). It also increased the activity of the liver GST enzyme in SD rats that were not induced with DMBA. CXBCH administration also increased the GST enzyme activity in SD rats induced with DMBA at 2 × 20 or 10 × 20 mg/kg BW. Observation of liver GST enzyme activity at the 30th week of treatment proved that DMBA induction decreased liver GST enzyme activity and CXBCH administration increased it. The GST enzyme activity of the DMBA group was lower than that of the standard group (*p* < 0.05), while the treatment groups that received CXBCH, thymoquinone, and tamoxifen showed higher GST activity than the DMBA group (*p* < 0.05). 

### 3.5. CXBC Preparations Increase the Number of CD4, CD8 and CD4CD25

Table 8 presents the results of the flow cytometry examination. It can be seen that DMBA induction at 10 × 20 mg/kg BW decreased absolute CD4 and CD4CD25 counts as the counts in the DMBA group was lower than those in the standard group (*p* < 0.05), with only a third. The absolute CD4CD25 count in the DMBA group was also lower (*p* < 0.05), but the ratio of CD4CD25 to CD4 in the DMBA group was higher than that in the standard group (*p* < 0.05).

CXBCH administration increased the absolute number of CD4 and CD4CD25 but decreased the percentage of CD4CD25/CD4. The CD4 and CD4CD25 counts in the CXBCH group were higher than those in the DMBA group (*p* < 0.05), but the CD4CD25/CD4 percentage in the CXBCH group was lower (*p* < 0.05). Among the treatment groups, the CXBCH2 group had the highest absolute numbers of CD4 and CD4CD25, followed by the CXBCH3 group and CXBCH1. The mean absolute CD4 count of the CXBCH2 group was almost the same as that of the thymoquinone group (*p* > 0.05). CXBCH administration for two weeks before and five weeks during DMBA induction reduced DMBA’s immunotoxic effect on CD4 and CD4CD25 counts. The CXBCH’s ability to inhibit the immunotoxic effects of DMBA was equivalent to that of thymoquinone at a dose of 50 mg/kg (*p* > 0.05).

### 3.6. CXBCH Increases Absolute CD8 and CD8CD25 Counts

Table 9 presents the absolute number of CD8 and CD8CD25 and the percentage of CD8CD25/CD8. It shows that DMBA induction reduced the absolute number of CD8 and CD8CD25, namely to ¼ of standard CD8 number and 3/5 of standard CD4CD25 number, but increased the percentage of CD8CD25 to CD8 (*p* < 0.05), although the percentage was still higher than the DMBA group (*p* < 0.05). 

CXBCH administration to DMBA-induced SD rats increased the CD8 and CD8CD25 counts but decreased the percentage of CD8CD25 to CD8. Total CD8 and CD8CD25 were found to be higher in the CXBCH group than those in the DMBA group (*p* < 0.05), but the ratio of CD8CD25 to CD8 was lower (*p* < 0.05). 

## 4. Discussion

This study aimed to determine the effectiveness of CXBCH preparations as chemopreventive, antioxidant, and immunomodulator in DMBA-induced SD rats. The novelty in this publication is the test material in the form of herbal honey preparations (CXBCH) containing Curcuma xanthorriza extract(CXE) and black cumin extract (BCE).

### 4.1. CXBCH Preparation Ingredients and Activities

Thymoquinone and curcumin are the main active substances in the CXBCH preparations. According to the results of the analysis of the CXBCH’s active ingredients, in addition to thymoquinone and curcumin, CXBCH preparations also contain fructose, eremantin, meglutol, monoolein, tur-meron, and palmitin. Thymoquinone is BC’s main active substance while curcumin is CX’s active substance. The composition of the active substances in BC and CX extracts is determined by the extraction method, the type of solvent compound, and the region of origin [62]. Making CXBCH preparations by utilizing honey as a solvent and flavoring medium can overcome the weaknesses of BC oil preparations, which often cause burping, an unattractive taste, and a pungent aroma [63,64]. The high levels of thymoquinone and curcumin in CXBCH preparations, accompanied with a pleasing sweet taste, indicates that the CXBC herbal honey preparations are in line with the expectations of both researchers and consumers [44,65].

Thymoquinone and curcumin have been shown to have various biological activities [33,66]. Nigelon is a polymer form of thymoquinone that inhibits the activity of cyclooxygenase and lipoxygenase enzymes in arachidonic metabolism; hence, it is believed that it can be employed as an analgesic, anti-allergic, anti-inflammatory, and anticancer agent [67]. Thymoquinone has also been demonstrated to be hepatoprotective [68], antioxidative [69], neuroprotective due to ischemia [70], antihyperlipidemic [71], nephroprotective [72], immunomodulatory by inhibiting NFkB [73], anti-autoimmune disease agent [74], and anti-cancer [75]. 

### 4.2. CXBCH Chemopreventive Activity

The research data showed that DMBA induction at 10 × 20 mg/kgBW resulted in nodule formation and carcinogenesis. Administration of CXBCH preparations, thymoquinone, and tamoxifen has been shown to inhibit such formation as the results showed that the formation and the number of nodules in the CXBCH, thymoquinone, and tamoxifen groups were lower than those in the DMBA group. This study is in line with the activity of thymoquinone and curcumin as antioxidants and anti-inflammatories, thereby reducing the formation of the active DMBA metabolite (DMBA-DE) [76,77].

The CXBCH content is thought to inhibit the meeting of AhR with DMBA (ligand), as there is no activation of the signal transduction pathway of AhR, and no active metabolite of DMBA-DE is formed [34,54,78]. Thymoquinone, dithymoquinone, dihydro-thymoquinone, unsaturated fatty acids, and sitosterol are compounds, with a molecular structure similar to AhR ligands [55], can act as AhR ligands as partial antagonists/agonists and are competitive against DMBA [56]. Active CXBCH preparations, such as polyphenols, flavonoids, curcumin, and thymoquinone, can competitively block the junction of DMBA with AhR, preventing the formation of the DMBA-AhR complex and preventing AhR receptor activation. There are insufficient cytochrome CYP1A1/CYP1B1 enzymes for the metabolism of DMBA to DMBA-DE because the ligand–receptor complex (DMBA-AhR) does not form, preventing AhR from translocating as a transcription factor and preventing the transcription of the CYP1A1/CYP1B1 gene [57,79]. Thymoquinone, flavone, and epigallocatechin (EPGK) activity of several scavenger substances has been compared [80]. Thymoquinone’s ability to scavenge or neutralize free radicals in skin is comparable to that of the polyphenol molecule found in tea [58,81].

### 4.3. CXBC Antioxidant Activity through Increased GST Expression and Decreases NO Levels

The results showed that CXBCH preparations decreased NO levels and increased GST levels. DMBA induction has been shown to increase plasma NO levels as the level was higher in the DMBA group than that in the standard and solvent groups (*p* < 0.05). Genotoxic stress is the cells’ response to the presence of DNA-damaging agents both from extracellular and intracellular sources, such as NO [82]. It can cause genetic changes and cell damage [59,83]. Mammalian cells have biochemical components as a defense system to maintain cell integrity from stressors both inside and outside the cells, including the antioxidant cytoprotective enzyme GST (Phase II) [17,84]. CXBCH preparations, such as thymoquinone and tamoxifen, could reduce NO levels and increase GST activity in DMBA-induced SD rats. The antioxidant mechanism of CXBCH can be explained by the results of this study which showed that DMBA induction decreased GST enzyme activity, while CXBCH administration before and during DMBA induction increased GST enzyme activity. The biochemical content of CXBCH appears to work directly in increasing the production of the GST enzyme [85]. These results align with those of previous studies, which have proven that the bioactive content of BC shows activity as a phase II enzyme promoter in both in vitro and in vivo tests [61,86,87]. The data from this study and the evidence from previous studies show that the antioxidant activity of CXBCH and thymoquinone is a promoter of GST gene activation, so the production of GST enzymes increases [84]. As a scavenger, thymoquinone and other active CXBCH substances can bind directly to the reactive radical, DMBA-DE, formed from phase I metabolism so that it is not reactive [35]. The rapid reaction between thymoquinone and GSH produces a reduced compound glutathione dihydro-thymoquinone (GDHTQ), whereas the slow reaction of thymoquinone with NADH and NADPH produces a reduced compound dihydro-thymoquinone (DHTQ) [88]. The antioxidant activity of DHTQ and GDHTQ as scavengers against active organic radicals (DPPH) is the same, while the ability of thymoquinone is lower [35,89].

### 4.4. CXBCH as Immunomodulator

Research has shown that DMBA induction causes oxidative stress and is immunosuppressive, as evidenced by a decrease in the number of leukocytes and lymphocytes and a decrease in the activity of GST enzymes in the liver and spleen. Administration of CXBCH preparations has been shown to increase the cellular components of blood and the number of lymphocytes. The CXBCH groups had higher CD4, CD8, and CD4CD25 cells than the DMBA group (*p* < 0.05). The results of this study are in accordance with those of previous studies, which show that DMBA and other xenobiotic PAHs result in the formation of reactive radicals that are immunotoxic [22,90], while BC and CX increase immune responses or are immunostimulant and antioxidative [91,92,93]. Curcumin has been shown to influence CD4Th differentiation in vivo [94]. Like curcumin, thymoquinone has increased the number of CD4Th lymphocytes in vivo [67,95]. Thymoquinone has been shown to increase macrophage activity by activating Toll-like receptors (TLRs) [68,96]. Thymoquinone and other active substances from Nigella sativa have been shown to increase lymphocyte proliferative activity, macrophage activity, and IFN-γ production in vivo [69,95,97].

Although it has been anticipated, this research still has some weaknesses. Due to technical limitations and problems, the researchers did not measure DMBA-adduct as a biomarker of genotoxic stress due to DMBA exposure. However, this weakness has been anticipated by the existence of the standard and solvent control groups.

## 5. Conclusions

CXBCH equivalent doses of 5, 10, and 15 mL/70kgBW had a chemopreventive effect in SD rats induced with DMBA at 10 × 20 mg/kgBW. The chemopreventive mechanism of CXBCH is as a blocking agent by blocking the initiation process by inhibiting the carcinogenesis process.

CXBC antioxidant activity and mechanism decrease serum NO levels and increase liver and spleen GST enzyme activity. As an immunomodulator, CXBCH preparations increase the number of CD4, CD8, and CD4CD25 lymphocytes.

## Figures and Tables

**Table 1:** Results of observations of the survival ability of each group of SD rats receiving CXBCH two weeks before and five weeks during DMBA induction.

Test Groups	n	Dead Beginning	Livability of Test Animals (%) Week			Percentage of Total Deaths (%)
			16	20	30	
Normal	10	1	90.00	90.00	90.00	10.00
CXBCH 1	10	1	90.00	90.00	90.00	10.00
CXBCH 2	10	2	80.00	80.00	80.00	20.00
CXBCH 3	10	1	90.00	90.00	80.00	20.00
Thymoquinone	10	1	90.00	90.00	90.00	10.00
Tamoxifen	10	2	80.0	80.00	70.00	30.00
DMBA	10	3	70.00	40.00	30.00	100.00
Solvent	10	1	90.00	90.00	90.00	10.00

**Table 2:** Routine blood test results of DMBA-induced SD rats and CXBCH treatment two weeks before and five weeks during induction. Blood sampling was carried out at the 30th week of the experiment.

Group	Leukocyte Count (×103/µL) (Mean ± sd)	Erythrocyte Count (×106/µL) (Mean ± sd)	Platelet Count (×103/µL) (Mean ± sd)	Hb Level (Mean ± sd)	MCV (Mean ± sd)	MCH (Mean ± sd)
Normal (10)	6.33 ± 1.37 *	8.32 ± 0.40^b,c,^*	981.33 ± 95.37 *	14.67 ± 0.52 *	58.33 ± 1.37 *	20.00 ± 0.89 *
CXBCH 1 (10)	9.86 ± 0.90 ^a^*	7.87 ± 0.27 *	668.71 ± 50.91 ^a,^*	14.74 ± 0.24 *	56.86 ± 0.89 ^a,^*	18.71 ± 0.49 ^a,^*
CXBCH 2 (10)	6.29 ± 0.49 *	7.35 ± 0.28 ^a^*	724.71 ± 181.25 *	14.44 ± 0.80 *	59.29 ± 0.49 *	19.29 ± 0.49 *
CXBCH 3 (10)	7.00 ± 0.93 *	7.65 ± 0.14 ^a^*	802.00 ± 106.23 *	14.59 ± 0.85 *	59.38 ± 0.52 *	19.38 ± 0.52 *
Thymoquinone (10)	6.86 ± 0.90 *	6.53 ± 0.94 ^a^*	734.29 ± 151.14 *	13.34 ± 0.74 *	57.34 ± 2.30 *	21.17 ± 1.21 *
Tamoxifen (10)	7.00 ± 2.68 *	6.59 ± 0.21 ^a^*	889.17 ± 387.24 *	13.83 ± 2.99 *	57.33 ± 1.63 *	19.67 ± 0.52 *
DMBA (10)	2.80 ± 1.10 ^a,b,c^	4.18 ± 0.94 ^a,b,c^	255.00 ± 70.31 ^a,b,c^	6.60 ± 2.07 ^a,b,c^	54.40 ± 1.52 ^a,b,c^	16.33 ± 0.52 ^a,b,c^
Solvent (10)	6.67 ± 1.03 *	9.14 ± 0.42 *	908.00 ± 120.10 *	15.67 ± 0.52 *	56.67 ± 1.37 ^a,^*	19.33 ± 0.52 *

Note: ^a^ = *p* < 0.05 for the normal group; ^b^ = *p* < 0.05 for thymoquinone group; ^c^ = *p* < 0.05 for Tamoxifen group; * = *p* < 0.05 for the DMBA group.

**Table 3:** Results of examination of blood urea and creatinine levels of DMBA-induced SD rats after receiving CXBCH treatment two weeks before and five weeks during DMBA induction. Blood sampling was carried out at the 30th week of the experiment.

Test Group	Serum Urea Level (Mean ± sd) (mg/dL)	Serum Creatinine Level (Mean ± sd) (mg/dL)	The Average Level of SGPT (Mean ± sd) (U/L)	Average Serum SGOT Level (Mean ± sd) (U/L)
Normal (10)	26.33 ± 1.37 ^c,^*	0.40 ± 0.00 ^b,c,^*	44.00 ± 0.89 ^c,^*	130.33 ± 1.37 ^c,^*
CXBCH 1 (10)	27.14 ± 4.81 *	0.26 ± 0.05 ^a,b,c,^*	52.00 ± 4.86 ^a,b,^*	107.19 ± 21.78 ^a,c,^*
CXBCH 2 (10)	30.29 ± 2.06 ^a,^*	0.30 ± 0.00 ^a,b,c,^*	58.00 ± 11.65 ^a,^*	88.00 ± 4.89 ^a,b,c,^*
CXBCH 3 (10)	33.63 ± 4.17 ^a,^*	0.30 ± 0.00 ^a,b,c,^*	55.38 ± 9.04 ^a,^*	83.50 ± 3.70 ^a,b,c,^*
Thymoquinone (10)	31.11 ± 7.26 *	0.34 ± 0.05 ^a,^*	44.31 ± 12.81 *	124.14 ± 5.46 ^a,c,^*
Tamoxifen (10)	37.00 ± 6.39 ^a^	0.35 ± 0.08 ^a,^*	95.33 ± 74.29 ^a,^*	206.17 ± 2.43 ^a,b,^*
DMBA(10)	40.80 ± 0.84 ^a,b,c^	0.54 ± 0.05a ^a,b,c^	156.80 ± 50.58 ^a,b,c^	830.40 ± 92.66 ^a,b,c^
Solvent (10)	35.00 ± 4.73 *	0.33 ± 0.05 ^a,^*	60.33 ± 7.23 *	92.00 ± 8.80 *

Note: ^a^ = *p* < 0.05 for the normal group; ^b^ = *p* < 0.05 for thymoquinone group; ^c^ = *p* < 0.05 for Tamoxifen group; * = *p* < 0.05 for the DMBA group.

**Table 4:** The examination results of the number of nodules in DMBA-induced female SD rats receiving CXBCH treatment two weeks before and five weeks during induction.

Test Group (n)	Incidence of Tumor Formation (%)	Number of Nodules Formed	Tumor Multiplicity (Nodule/Rat)	Total Weight of Nodules (g)
Normal (10)	0	0	0.0 ± 0.0	0
CXBCH 1 (10)	50%	8	0.50 ± 0.50	2.51
CXBCH 2 (10)	50%	8	0.80 ± 0.92	3.25
CXBCH 3 (10)	50%	6	0.73 ± 0.79	4.17
Thymoquinone (10)	30%	3	0.30 ± 0.48	1.20
Tamoxifen (10)	30%	5	0.46 ± 0.93	1.40
DMBA (10)	100%	14	1.40 ± 1.1	10.53
Solvent (10)	0	0	0.0 ± 0.0	0

**Table 5:** Histopathological examination of mammary tissue with or without mammary tissue tumor nodules in SD rats induced with DMBA and receiving CXBCH treatment two weeks before and five weeks during induction. Tissue collection was carried out at week 30.

Test Groups	Type of Histopathological Picture (%)			% Inhibition of ACM
	TAP	PP	ACM (Invasive)	
Normal (10)	100.00	0	0	-
CXBCH 1 (10)	50.00	30.00	20.00	80
CXBCH 2 (10)	60.00	10.00	30.00	70
CXBCH 3 (10)	55.00	40.00	10.00	90
Thymoquinone (10)	70.00	30.00	0	100
Tamoxifen (10)	72.70	0	27.30	73
DMBA (10)	0	0	100.00	0
Solvent (10)	100	0	0	0

Notes: TAP = no change (normal); PP = proliferation; ACM = adenocarcinoma.

**Table 6:** Serum NO levels in SD rats treated with CXBCH two weeks prior to and five weeks during DMBA induction at week 30.

Test Group	Average Serum NO Level (µM) (Mean ± sd)
Normal (10)	0.20 ± 0.07 ^b,c,^*
CXBCH 1 (10)	0.21 ± 0.05 ^a,b,c,^*
CXBCH 2 (10)	0.24 ± 0.05 ^a,b,c,^*
CXBCH 3 (10)	0.18 ± 0.12 ^a,b,c,^*
Thymoquinone (10)	0.29 ± 0.09 ^a,c,^*
Tamoxifen (10)	0.23 ± 0.01 ^a,b,^*
DMBA (10)	0.38 ± 0.09 ^a,b,c^
Solvent (10)	0.18 ± 0.02 ^a,b,c,^*

Note: ^a^ = *p* < 0.05 for the normal group; ^b^ = *p* < 0.05 for thymoquinone group; ^c^ = *p* < 0.05 for Tamoxifen group; * = *p* < 0.05 for the DMBA group.

**Table 7:** GST activity of the liver and spleen of SD rats that received CXBCH two weeks before and five weeks during DMBA induction at week 30.

Group	GST Activity in Liver and Spleen Tissue (Mean ± sd) (ug/min/mL)	
	Spleen	Liver
Normal (10)	8.70 ± 0.89 *	82.91 ± 7.93 *
CXBCH 1 (10)	17.39 ± 2.17 ^a,^*	106.98 ± 5.45 ^a,^*
CXBCH 2 (10)	18.87 ± 1.30 ^a,^*	112.21 ± 8.87 ^a,^*
CXBCH 3 (10)	20.44 ± 0.98 ^a,^*	113.83 ± 10.08 ^a,^*
Thymoquinone (10)	18.74 ± 2.38 ^a,^*	91.14 ± 7.18 ^a,^*
Tamoxifen (10)	17.62 ± 2.61 ^a,^*	83.29 ± 11.14 *
DMBA (10)	6.86 ± 0.91 ^a^	65.54 ± 3.31 ^a^
Solvent (10)	8.41 ± 0.76 *	83.50 ± 7.31 *

Note.: ^a^ = *p* < 0.05 for the normal group; * = *p* < 0.05 for the DMBA group.

**Table 8:** Examination results of the absolute number of CD4 lymphocytes in the peripheral blood of SD rats induced with DMBA at 2 × 20 mg/kg/week for five weeks after receiving seven weeks of CXBCH treatment. Blood sampling was carried out at the 30th week of the experiment.

Test Group	Absolute Amount CD4 (Mean ± SD)	Absolute CD4CD25 Count (Mean ± SD)	Percentage of CD4CD25 to CD4 (Mean ± SD)
Normal (10)	1575.67 ± 131.70 *	70.50 ± 11.76	4.41 ± 0.01 ^b,c,^*
CXBC 1 (10)	1619.57 ± 519.86 *	80.86 ± 17.78 *	4.19 ± 0.01 *
CXBC 2 (10)	1868.57 ± 382.55 *	113.32 ± 20.58 ^a,^*	6.14 ± 0.01 ^a,b,c,^*
CXBC 3 (10)	1668.75 ± 398.01 *	92.50 ± 20.53 *	5.66 ± 0.01 ^a,^*
Thymoquinone (10)	1799.83 ± 429.90 *	97.50 ± 21.69 *	5.62 ± 0.02 ^a,^*
Tamoxifen (10)	1940.00 ± 203.76 ^a^*	84.50 ± 13.46 *	4.34 ± 0.00 ^a,b,^*
DMBA (10)	484.17 ± 33.98 ^a,b,c^	45.17 ± 9.07 ^a,b,c^	11.50 ± 0.02 ^a,b,c^
Solvent (10)	1490.33 ± 508.98 *	109.33 ± 64.06 *	7.35 ± 0.04 ^a,b,c,^*

Note. ^a^ = *p* < 0.05 for the normal group; ^b^ = *p* < 0.05 for thymoquinone group; ^c^ = *p* < 0.05 for Tamoxifen group; * = *p* < 0.05 for the DMBA group.

**Table 9:** The absolute number of peripheral blood CD8 lymphocytes of SD rats induced with DMBA at 2 × 20 mg/kgBW/week for five weeks after receiving CXBCH treatment for seven weeks. Blood sampling was carried out at the 30th week of the experiment.

Test Group	Absolute CD8 Count (Mean ± SD)	The Absolute Number of CD8CD25 (Mean ± SD)	Percentage of CD8CD25 to CD8 (Mean ± SD)
Normal (10)	580.00 ± 66.63 ^b,c,^*	52.50 ± 9.39 *	9.00 ± 1.30 *
CXBCH 1 (10)	840.43 ± 48.64 ^a,c,^*	77.00 ± 2.15 ^a,^*	10.70 ± 3.67 *
CXBCH 2 (10)	860.00 ± 33.95 ^a,b,c,^*	85.28 ± 2.6 ^a,b,^*	10.84 ± 3.00 *
CXBCH 3 (10)	512.50 ± 47.42 ^b,c,^*	53.00 ± 3.86 *	13.18 ± 3.72 *
Thymoquinone (10)	813.33 ± 18.17 ^a,c,^*	67.16 ± 16.77 *	8.70 ± 3.09 *
Tamoxifen (10)	915.00 ± 17.13 ^a,b,^*	68.16 ± 20.62 *	7.52 ± 2.32 *
DMBA (10)	137.00 ± 18.48 ^a,b,c^	32.66 ± 6.43 ^a,b,c^	23.75 ± 2.5 ^a,b,c^
Solvent (10)	668.33 ± 39.56 ^a,b,c,^*	59.33 ± 16.94 ^b,^*	10.74 ± 5.6 *

Note: ^a^ = *p* < 0.05 for the normal group; ^b^ = *p* < 0.05 for thymoquinone group; ^c^ = *p* < 0.05 for Tamoxifen group; * = *p* < 0.05 for the DMBA group.

## Data Availability

Not applicable.

## Supplementary Materials

The following supporting information can be downloaded at: https://www.mdpi.com/article/10.3390/nu15020371/s1, Table S1: Phytochemical analysis of CXE, BCE, and CXBCH preparations. Table S2: Profile volatile Compounds of CXBCH preparations, CXE, and BCE from the GCMS examination. Table S3: Formula, molecule name, RT, annotation delta mass and max area (absolute) observed with LC-HRMS from CXBCH preparations. Figure S1: GCMS analysis of CXBCH preparations. Figure S2: The total ion chromatogram of the CXBCH preparation was observed using LC-HRMS. Figure S3: Nodules (arrows) were formed in the mammary gland in the 20th week of observation of SD rats after receiving CXBCH 2 weeks before and five weeks after DMBA induction. Nodules formed in all parts of the mammary gland (fore legs (A), near the hind legs (B), and some nodules break up (C). Note: Arrows indicate nodules.

## References

[1] Lee L.L., Lee J.S.C., Waldman S.D., Casper R.F., Grynpas M.D. (2002). Polycyclic aromatic hydrocarbons present in cigarette smoke cause bone loss in an ovariectomized rat model. Bone.

[2] Kerdelhué B., Forest C., Coumoul X. (2016). Dimethyl-Benz(a)anthracene: A mammary carcinogen and a neuroendocrine disruptor. Biochim. Open.

[3] De Oliveira K.D., Avanzo G.U., Tedardi M.V., Rangel M.M.M., Avanzo J.L., Fukumasu H., Rao K.V.K., Sinhorini I.L., Dagli M.L.Z. (2015). Chemical carcinogenesis by DMBA (7,12-dimethylbenzanthracene)in female BALB/c mice: New facts. Braz. J. Vet. Res. Anim. Sci..

[4] Gopalakrishnan T., Ganapathy S., Veeran V., Namasivayam N. (2019). Preventive effect of D-carvone during DMBA induced mouse skin tumorigenesis by modulating xenobiotic metabolism and induction of apoptotic events. Biomed. Pharmacother..

[5] Reitsma M.B., Kendrick P.J., Ababneh E., Abbafati C., Abbasi-Kangevari M., Abdoli A., Abedi A., Abhilash E.S., Abila D.B., Aboyans V. (2021). Spatial, temporal, and demographic patterns in prevalence of smoking tobacco use and attributable disease burden in 204 countries and territories, 1990–2019: A systematic analysis from the Global Burden of Disease Study 2019. Lancet.

[6] Crosby A., Dunn J.L., Aditjondro E. (2019). Rachfiansyah Tobacco Control Is a Wicked Problem: Situating Design Responses in Yogyakarta and Banjarmasin. She Ji.

[7] Ahsan A. (2014). Bunga Rampai Fakta Tembakau dan Permasalahannya di Indonesia 2014.

[8] Hua M., Talbot P. (2016). Potential health effects of electronic cigarettes: A systematic review of case reports. Prev. Med. Rep..

[9] Forster M., McAughey J., Prasad K., Mavropoulou E., Proctor C. (2018). Assessment of tobacco heating product THP1.0. Part 4: Characterisation of indoor air quality and odour. Regul. Toxicol. Pharmacol..

[10] Control D., Cdc P. (2021). GATS (Global Adult Tobacco Survey) Comparison Fact Sheet, Indonesia 2011 and 2021. https://cdn.who.int/media/docs/default-source/ncds/ncd-surveillance/data-reporting/indonesia/indonesia-national-2021-factsheet.pdf?sfvrsn=53eac4fd_1&download=true.

[11] Almeida-da-Silva C.L.C., Matshik Dakafay H., O’Brien K., Montierth D., Xiao N., Ojcius D.M. (2020). Effects of electronic cigarette aerosol exposure on oral and systemic health. Biomed. J..

[12] Xi B., Liang Y., Liu Y., Yan Y., Zhao M., Ma C., Bovet P. (2016). Tobacco use and second-hand smoke exposure in young adolescents aged 12–15 years: Data from 68 low-income and middle-income countries. Lancet Glob. Health.

[13] Zhang X., Zhang L., Yang L., Zhou Q., Xing W., Toriba A. (2020). Characteristics of Polycyclic Aromatic Hydrocarbons (PAHs) and Common Air Pollutants at Wajima, a Remote Background Site in Japan. Int. J. Environ. Res. Public Health.

[14] Meckley D.R., Hayes J.R., Van Kampen K.R., Ayres P.H., Mosberg A.T., Swauger J.E. (2004). Comparative study of smoke condensates from 1R4F cigarettes that burn tobacco versus ECLIPSE cigarettes that primarily heat tobacco in the SENCAR mouse dermal tumor promotion assay. Food Chem. Toxicol..

[15] Septiono W., Kuipers M.A.G., Ng N., Kunst A.E. (2020). Changes in adolescent smoking with implementation of local smoke-free policies in Indonesia: Quasi-experimental repeat cross-sectional analysis of national surveys of 2007 and 2013. Drug Alcohol Depend..

[16] Miyata M., Furukawa M., Takahashi K., Gonzalez F.J., Yamazoe Y. (2001). Mechanism of 7,12-dimethylbenz[a]anthracene-induced immunotoxicity: Role of metabolic activation at the target organ. Jpn. J. Pharmacol..

[17] Galván N., Page T.J., Czuprynski C.J., Jefcoate C.R. (2006). Benzo(a)pyrene and 7,12-dimethylbenz(a)anthrecene differentially affect bone marrow cells of the lymphoid and myeloid lineages. Toxicol. Appl. Pharmacol..

[18] Pugalendhi P., Manoharan S., Panjamurthy K., Balakrishnan S., Nirmal M.R. (2009). Antigenotoxic effect of genistein against 7,12-dimethylbenz[a]anthracene induced genotoxicity in bone marrow cells of female Wistar rats. Pharmacol. Rep..

[19] Chełchowska M., Ambroszkiewicz J., Gajewska J., Mazur J., Lewandowski L., Reśko-Zachara M., Maciejewski T.M. (2018). Influence of active exposure to Tobacco smoke on nitric oxide status of pregnant women. Int. J. Environ. Res. Public Health.

[20] Soares R., Costa C. (2009). Oxidative Stress, Inflammation and Angiogenesis in the Metabolic Syndrome.

[21] Abdel-Wahhab M.A., Aljawish A., El-Nekeety A.A., Abdel-Aiezm S.H., Abdel-Kader H.A.M., Rihn B.H., Joubert O. (2015). Chitosan nanoparticles and quercetin modulate gene expression and prevent the genotoxicity of aflatoxin B1 in rat liver. Toxicol. Rep..

[22] Gao J., Mitchell L.A., Lauer F.T., Burchiel S.W. (2008). p53 and ATM/ATR regulate 7,12-dimethylbenz[a]anthracene-induced immunosuppression. Mol. Pharmacol..

[23] Gao J., Lauer F.T., Mitchell L.A., Burchiel S.W. (2007). Microsomal expoxide hydrolase Is required for 7,12-dimethylbenz[a]anthracene (DMBA)—Induced immunotoxicity in mice. Toxicol. Sci..

[24] Sun X., Bernhardt S.M., Glynn D.J., Hodson L.J., Woolford L., Evdokiou A., Yan C., Du H., Robertson S.A., Ingman W.V. (2021). Attenuated TGFB signalling in macrophages decreases susceptibility to DMBA-induced mammary cancer in mice. Breast Cancer Res..

[25] Fujii S.I., Shimizu K., Shimizu T., Lotze M.T. (2001). Interleukin-10 promotes the maintenance of antitumor CD8+ T-cell effector function in situ. Blood.

[26] Mattes J., Hulett M., Xie W., Hogan S., Rothenberg M.E., Foster P., Parish C. (2003). Immunotherapy of cytotoxic T cell-resistant tumors by T helper 2 cells: An eotaxin and STAT6-dependent process. J. Exp. Med..

[27] Tarigan S.P., Soeroso N.N., Tumanggor C.A.K., Gani S., Pradana A. (2019). Clinical profile of male patients with non-small cell lung cancer in Adam Malik General Hospital, Medan, Indonesia. Open Access Maced. J. Med. Sci..

[28] Anisimov V.N., Popovich I.G., Zabezhinski M.A., Anisimov S.V., Vesnushkin G.M., Vinogradova I.A. (2006). Melatonin as antioxidant, geroprotector and anticarcinogen. Biochim. Biophys. Acta Bioenerg..

[29] Kleiner H.E. (2001). Oral administration of naturally occurring coumarins leads to altered phase I and II enzyme activities and reduced DNA adduct formation by polycyclic aromatic hydrocarbons in various tissues of SENCAR mice. Carcinogenesis.

[30] Shimada T., Fujii-Kuriyama Y. (2004). Metabolic activation of polycyclic aromatic hydrocarbons to carcinogens by cytochromes P450 1A1 and 1B1. Cancer Sci..

[31] Kim H., Hall P., Smith M., Kirk M., Prasain J.K., Barnes S., Grubbs C. (2004). Chemoprevention by Grape Seed Extract and Genistein in Carcinogen-induced Mammary Cancer in Rats Is Diet Dependent. J. Nutr..

[32] Nugraha R.V., Ridwansyah H., Ghozali M., Khairani A.F., Atik N. (2020). Traditional Herbal Medicine Candidates as Complementary Treatments for COVID-19: A Review of Their Mechanisms, Pros and Cons. Evid. Based Complement. Altern. Med..

[33] Ahmad A., Mishra R.K., Vyawahare A., Kumar A., Rehman M.U., Qamar W., Khan A.Q., Khan R. (2019). Thymoquinone (2-Isoprpyl-5-methyl-1, 4-benzoquinone) as a chemopreventive/anticancer agent: Chemistry and biological effects. Saudi Pharm. J..

[34] Hidayati T., Akrom, Indrayanti, Sagiran (2019). Chemopreventive effect of black cumin seed oil (BCSO) by increasing p53 expression in dimethylbenzanthracene (DMBA)-induced Sprague Dawley rats. Res. J. Chem. Environ..

[35] Mansour M.A., Nagi M.N., El-Khatib A.S., Al-Bekairi A.M. (2002). Effects of thymoquinone on antioxidant enzyme activities, lipid peroxidation and dt-diaphorase in different tissues of mice: A possible mechanism of action. Cell Biochem. Funct..

[36] Wani M.R., Shadab G.G.H.A. (2021). Low doses of thymoquinone protect isolated human blood cells from TiO_2_ nanoparticles induced oxidative stress, hemolysis, cytotoxicity, DNA damage and collapse of mitochondrial activity. Phytomed. Plus.

[37] Fu Y.S., Chen T.H., Weng L., Huang L., Lai D., Weng C.F. (2021). Pharmacological properties and underlying mechanisms of curcumin and prospects in medicinal potential. Biomed. Pharmacother..

[38] Zhao G., Qi L., Wang Y., Li X., Li Q., Tang X., Wang X., Wu C. (2021). Antagonizing effects of curcumin against mercury-induced autophagic death and trace elements disorder by regulating PI3K/AKT and Nrf2 pathway in the spleen. Ecotoxicol. Environ. Saf..

[39] Oon S.F., Nallappan M., Tee T.T., Shohaimi S., Kassim N.K., Sa’ariwijaya M.S.F., Cheah Y.H. (2015). Xanthorrhizol: A review of its pharmacological activities and anticancer properties. Cancer Cell Int..

[40] Salleh N., Ismail S., Ab Halim M.R. (2016). Effects of Curcuma xanthorrhiza extracts and their constituents on phase II drug-metabolizing enzymes activity. Pharmacogn. Res..

[41] Singgih Wahono C., Diah Setyorini C., Kalim H., Nurdiana N., Handono K. (2017). Effect of Curcuma xanthorrhiza Supplementation on Systemic Lupus Erythematosus Patients with Hypovitamin D Which Were Given Vitamin D 3 towards Disease Activity (SLEDAI), IL-6, and TGF- β 1 Serum. Int. J. Rheumatol..

[42] Silva T.M.S., Santos F.P., Evangelista-Rodrigues A., da Silva E.M.S., da Silva G.S., de Novais J.S., de Assis Ribeiro dos Santos F., Camara C.A. (2013). Phenolic compounds, melissopalynological, physicochemical analysis and antioxidant activity of jandaíra (Melipona subnitida) honey. J. Food Compos. Anal..

[43] Seraglio S.K.T., Silva B., Bergamo G., Brugnerotto P., Gonzaga L.V., Fett R., Costa A.C.O. (2019). An overview of physicochemical characteristics and health-promoting properties of honeydew honey. Food Res. Int..

[44] Khan S.U., Anjum S.I., Ansari M.J., Khan M.H.U., Kamal S., Rahman K., Shoaib M., Man S., Khan A.J., Khan S.U. (2019). Antimicrobial potentials of medicinal plant’s extract and their derived silver nanoparticles: A focus on honey bee pathogen. Saudi J. Biol. Sci..

[45] Hidayati T., Indrayanti, Sagiran DMBA induction increases H-ras gene expression and decreases CD8 count in sprague dawley rats. Proceedings of the 2019 6th International Conference on Biomedical and Bioinformatics Engineering.

[46] Ibrahim A.B., Zaki H.F., Ibrahim W.W., Omran M.M., Shouman S.A. (2019). Evaluation of tamoxifen and simvastatin as the combination therapy for the treatment of hormonal dependent breast cancer cells. Toxicol. Rep..

[47] Fonseca T.G., Carriço T., Fernandes E., Abessa D.M.S., Tavares A., Bebianno M.J. (2019). Impacts of in vivo and in vitro exposures to tamoxifen: Comparative effects on human cells and marine organisms. Environ. Int..

[48] Yamanoshita O., Ichihara S., Hama H., Ichihara G., Chiba M., Kamijima M., Takeda I., Nakajima T. (2007). Chemopreventive effect of selenium-enriched Japanese radish sprout against breast cancer induced by 7,12-dimethylbenz[a]anthracene in rats. Tohoku J. Exp. Med..

[49] Panda V., Deshmukh A., Singh S., Shah T., Hingorani L. (2021). An Ayurvedic formulation of Emblica officinalis and Curcuma longa alleviates insulin resistance in diabetic rats: Involvement of curcuminoids and polyphenolics. J. Ayurveda Integr. Med..

[50] Soumaya K.J., Zied G., Nouha N., Mounira K., Kamel G., Genviève F.D.M., Leila G.C. (2014). Evaluation of in vitro antioxidant and apoptotic activities of Cyperus rotundus. Asian Pac. J. Trop. Med..

[51] Akanni O.O., Owumi S.E., Adaramoye O.A. (2014). In vitro studies to assess the antioxidative, radical scavenging and arginase inhibitory potentials of extracts from Artocarpus altilis, Ficus exasperate and Kigelia africana. Asian Pac. J. Trop. Biomed..

[52] Khazdair M.R., Gholamnezhad Z., Rezaee R., Boskabady M.H. (2021). A qualitative and quantitative comparison of Crocus sativus and Nigella sativa immunomodulatory effects. Biomed. Pharmacother..

[53] Noori S., Kiasat A.R., Kolahi M., Mirzajani R., Seyyed Nejad S.M. (2022). Determination of secondary metabolites including curcumin in Rheum ribes L. and surveying of its antioxidant and anticancer activity. J. Saudi Chem. Soc..

[54] Aryal B., Adhikari B., Aryal N., Bhattarai B.R., Khadayat K., Parajuli N. (2021). LC-HRMS Profiling and Antidiabetic, Antioxidant, and Antibacterial Activities of *Acacia catechu* (L.f.) Willd. Biomed. Res. Int..

[55] Dalhoumi W., Guesmi F., Bouzidi A., Akermi S., Hfaiedh N., Saidi I. (2022). Therapeutic strategies of *Moringa oleifera* Lam. (Moringaceae) for stomach and forestomach ulceration induced by HCl/EtOH in rat model. Saudi J. Biol. Sci..

[56] (2016). Majdalawieh AF & Fayyad MW Recent advances on the anti-cancer properties of *Nigella sativa*, a widely used food additive. J. Ayurveda Integr. Med..

[57] Roy A.M., Baliga M.S., Katiyar S.K. (2005). Epigallocatechin-3-gallate induces apoptosis in estrogen receptor-negative human breast carcinoma cells via modulation in protein expresssion of p53 and Bax and caspase-3 activation. Mol. Cancer Ther..

[58] Hamza A.A., Khasawneh M.A., Elwy H.M., Hassanin S.O., Elhabal S.F., Fawzi N.M. (2022). Salvadora persica attenuates DMBA-induced mammary cancer through downregulation oxidative stress, estrogen receptor expression and proliferation and augmenting apoptosis. Biomed. Pharmacother..

[59] Aydin M.S., Caliskan A., Kocarslan A., Kocarslan S., Yildiz A., Günay S., Savik E., Hazar A., Yalcin F. (2014). Intraperitoneal curcumin decreased lung, renal and heart injury in abdominal aorta ischemia/reperfusion model in rat. Int. J. Surg..

[60] Vaziri N.D., Ni Z., Oveisi F., Liang K., Pandian R. (2002). Enhanced nitric oxide inactivation and protein nitration by reactive oxygen species in renal insufficiency. Hypertension.

[61] Gholamnezhad Z., Boskabady M.H., Hosseini M. (2019). The effect of chronic supplementation of Nigella sativa on splenocytes response in rats following treadmill exercise. Drug Chem. Toxicol..

[62] Monton C., Settharaksa S., Luprasong C., Songsak T. (2019). An optimization approach of dynamic maceration of *Centella asiatica* to obtain the highest content of four centelloids by response surface methodology. Rev. Bras. Farmacogn..

[63] Aljohar H.I., Maher H.M., Albaqami J., Al-Mehaizie M., Orfali R., Orfali R., Alrubia S. (2018). Physical and chemical screening of honey samples available in the Saudi market: An important aspect in the authentication process and quality assessment. Saudi Pharm. J..

[64] Khan S.U., Anjum S.I., Rahman K., Ansari M.J., Khan W.U., Kamal S., Khattak B., Muhammad A., Khan H.U. (2018). Honey: Single food stuff comprises many drugs. Saudi J. Biol. Sci..

[65] Cianciosi D., Forbes-Hernández T.Y., Afrin S., Gasparrini M., Reboredo-Rodriguez P., Manna P.P., Zhang J., Lamas L.B., Flórez S.M., Toyos P.A. (2018). Phenolic compounds in honey and their associated health benefits: A review. Molecules.

[66] Hatipoglu D., Keskin E. (2022). The effect of curcumin on some cytokines, antioxidants and liver function tests in rats induced by Aflatoxin B1. Heliyon.

[67] Islam M.T., Khan M.R., Mishra S.K. (2019). An updated literature-based review: Phytochemistry, pharmacology and therapeutic promises of *Nigella sativa* L.. Orient. Pharm. Exp. Med..

[68] Akrom A., Darmawan E., Yuhelvia L. (2015). Black Cumin Seed Oilas Hepatoprotector in Decreasing SGPT and SGOT Activity and Increasing p53 Gene Expression in Sprague Dawley Rats Induced by Alloxan. Int. J. Public Health Sci..

[69] Burits M., Bucar F. (2000). Antioxidant activity of *Nigella sativa* essential oil. Phyther. Res..

[70] Çelik F., Göçmez C., Karaman H., Kamaşak K., Kaplan I., Akil E., Tufek A., Guzel A., Uzar E. (2014). Therapeutic Effects of Thymoquinone in a Model of Neuropathic Pain. Curr. Ther. Res. Clin. Exp..

[71] Farkhondeh T., Samarghandian S., Borji A. (2017). An overview on cardioprotective and anti-diabetic effects of thymoquinone. Asian Pac. J. Trop. Med..

[72] Shaterzadeh-Yazdi H., Noorbakhsh M.-F., Samarghandian S., Farkhondeh T. (2018). An Overview on Renoprotective Effects of Thymoquinone. Kidney Dis..

[73] Umar S., Shah M.A.A., Munir M.T., Yaqoob M., Fiaz M., Anjum S., Kaboudi K., Bouzouaia M., Younus M., Nisa Q. (2016). Synergistic effects of thymoquinone and curcumin on immune response and anti-viral activity against avian influenza virus (H9N2) in turkeys. Poult. Sci..

[74] Ali M.Y., Akter Z., Mei Z., Zheng M., Tania M., Khan M.A. (2021). Thymoquinone in autoimmune diseases: Therapeutic potential and molecular mechanisms. Biomed. Pharmacother..

[75] Begum S., Mannan A. (2020). A Review on Nigella sativa: A Marvel Herb. J. Drug Deliv. Ther..

[76] Rajkamal G., Suresh K., Sugunadevi G., Vijayaanand M.A., Rajalingam K. (2010). Evaluation of chemopreventive effects of Thymoquinone on cell surface glycoconjugates and cytokeratin expression during DMBA induced hamster buccal pouch carcinogenesis. BMB Rep..

[77] Singletary K., MacDonald C., Iovinelli M., Fisher C., Wallig M. (1998). Effect of the β-diketones diferuloylmethane (curcumin) and dibenzoylmethane on rat mammary DNA adducts and tumors induced by 7,12-dimethylbenz[a]anthracene. Carcinogenesis.

[78] Yeh H.C. (1999). and G.; Cellular The flavonoid galangin is an inhibitor of CYP1A1 activity and an agonist/antagonist of the aryl hydrocarbon receptor. Br. J. Cancer.

[79] Jensen B.A., Leeman R.J., Schlezinger J.J., Sherr D.H. (2003). Aryl hydrocarbon receptor (AhR) agonists suppress interleukin-6 expression by bone marrow stromal cells: An immunotoxicology study. Environ. Health Glob. Access Sci. Source.

[80] Lutterodt H., Luther M., Slavin M., Yin J.J., Parry J., Gao J.M., Yu L.L. (2010). Fatty acid profile, thymoquinone content, oxidative stability, and antioxidant properties of cold-pressed black cumin seed oils. LWT—Food Sci. Technol..

[81] Abbas F., Eladl M.A., El-Sherbiny M., Abozied N., Nabil A., Mahmoud S.M., Mokhtar H.I., Zaitone S.A., Ibrahim D. (2022). Celastrol and thymoquinone alleviate aluminum chloride-induced neurotoxicity: Behavioral psychomotor performance, neurotransmitter level, oxidative-inflammatory markers, and BDNF expression in rat brain. Biomed. Pharmacother..

[82] El Aziz M.A., Hassan H.A., Mohammed M.H., Meki A. (2005). The biochemical and morphological alterations following administration of melatonin, retinoic acid and Nigella sativa in mammary carcinoma:an animal model. Int. J. Exp. Pathol.

[83] Yuk-kwan C., Shui-sang H., Li-min L.I.N. (2002). The mRNA expression of inducible nitric oxide synthase in DMBA-induced hamster buccal-pouch carcinomas: An In Situ RT-PCR study. Int. J. Exp. Pathol..

[84] Alghamdi F., Al-Seeni M.N., Ghoneim M.A. (2020). Potential synergistic antioxidant effect of thymoquinone and vitamin E on cisplatin-induced acute nephropathy in rats. Clin. Nutr. Exp..

[85] Fusi J., Bianchi S., Daniele S., Pellegrini S., Martini C., Galetta F., Giovannini L., Franzoni F. (2018). An In Vitro comparative study of the antioxidant activity and SIRT1 modulation of natural compounds. Biomed. Pharmacother..

[86] Ramaswami G., Chai H., Yao Q., Lin P.H., Lumsden A.B., Chen C. (2004). Curcumin blocks homocysteine-induced endothelial dysfunction in porcine coronary arteries. J. Vasc. Surg..

[87] Tabassum S., Rosli N., Ichwan S.J.A., Mishra P. (2021). Thymoquinone and its pharmacological perspective: A review. Pharmacol. Res. Mod. Chin. Med..

[88] Kassab R.B., El-Hennamy R.E. (2017). The role of thymoquinone as a potent antioxidant in ameliorating the neurotoxic effect of sodium arsenate in female rat. Egypt. J. Basic Appl. Sci..

[89] Yaman I., Balikci E. (2010). Protective effects of nigella sativa against gentamicin-induced nephrotoxicity in rats. Exp. Toxicol. Pathol..

[90] Petroni G., Galluzzi L. (2021). Impact of treatment schedule on the efficacy of cytostatic and immunostimulatory agents. Oncoimmunology.

[91] Chai Y.S., Chen Y.Q., Lin S.H., Xie K., Wang C.J., Yang Y.Z., Xu F. (2020). Curcumin regulates the differentiation of naïve CD4+T cells and activates IL-10 immune modulation against acute lung injury in mice. Biomed. Pharmacother..

[92] Reddy P.S., Begum N., Mutha S., Bakshi V. (2016). Beneficial effect of Curcumin in Letrozole induced polycystic ovary syndrome. Asian Pac. J. Reprod..

[93] Salem M.L. (2005). Immunomodulatory and therapeutic properties of the Nigella sativa L. seed. Int. Immunopharmacol..

[94] Thimmulappa R.K., Mudnakudu-Nagaraju K.K., Shivamallu C., Subramaniam K.J.T., Radhakrishnan A., Bhojraj S., Kuppusamy G. (2021). Antiviral and immunomodulatory activity of curcumin: A case for prophylactic therapy for COVID-19. Heliyon.

[95] Shaterzadeh-Yazdi H., Noorbakhsh M.-F., Hayati F., Samarghandian S., Farkhondeh T. (2018). Immunomodulatory and Anti-inflammatory Effects of Thymoquinone. Cardiovasc. Hematol. Disord. Targets.

[96] Finlay T.M., Abdulkhalek S., Gilmour A., Guzzo C., Jayanth P., Amith S.R., Gee K., Beyaert R., Szewczuk M.R. (2010). Thymoquinone-induced Neu4 sialidase activates NFκB in macrophage cells and pro-inflammatory cytokines In Vivo. Glycoconj. J..

[97] Aljohani Z.M., Alharbi A.A., Alsaedi M., Alahmadi A.F., Alahmadi O.A., Alharbi A.A., Abo-Haded H.M. (2015). Evaluation of the potential beneficial effects of thymoquinone against nicotine induced toxicity. Int. J. Pharm. Clin. Res..

