# Supplementary material for: Herbal Honey Preparations of Curcuma Xanthorriza and Black Cumin Protect against Carcinogenesis through Antioxidant and Immunomodulatory Activities in Sprague Dawley (SD) Rats Induced with Dimethylbenz(a)anthracene
Source: Nutrients. 2023 Jan 11;15(2):371. doi: 10.3390/nu15020371 (PMC9867330; doi:10.3390/nu15020371)
Supplement: Supplementary file 1 [file nutrients-15-00371-s001.zip › nutrients-2064608-supplementary.pdf]

Supplementary material 1.

Table 1. Phytochemical analysis of CXE, BCE, and CXBCH preparations.

**Test**	**Samples and materials**		
	**CXE**	**BCE**	**CXBCH Preparation**
**Qualitative**			
Alkaloids	++	+	++
Flavonoids	++	++	++
Phenolic	++	++	++
Saponins	+	+	+
Triterpenoids	+	++	+
**Quantitative**			
Polyphenol	142.23 ppm	42.51ppm	38.87 ppm
Flavonoids	116.41 ppm	31.74 ppm	56.86 ppm
Thymoquinone	-	54.71 mg/g	46.45 mg/mL
Curcumin	62.28 mg/mL	-	68.86 mg/mL

Supplementary material 2

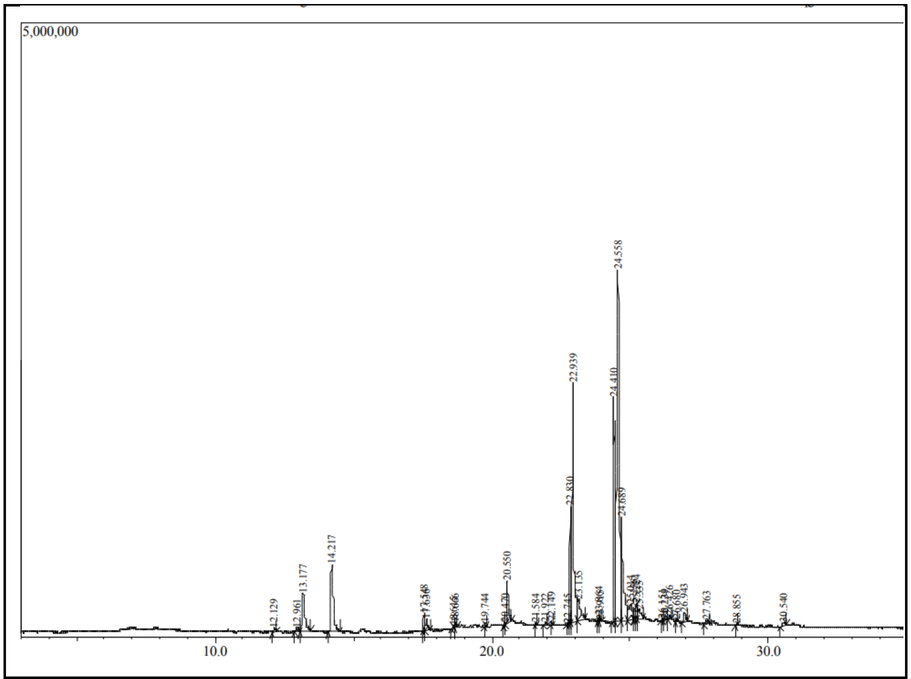

Fig 2. GCMS analysis of CXBCH preparations

Supplementary material 3

Table 2. profile volatile Compounds of CXBCH preparations, CXE, and BCE from the GCMS examination

No Peak	R.Time	I.Time	F.Time	Composition	Formula	Name
1	12.129	12.045	12.240	0.32	C15H24	1,6,10-dodecatriene, 7,11-dimethyl-3-methylene-, (e)-
2	12.961	12.870	13.080	0.32	C15H24	**gamma.-curcumene**
3	13.177	13.080	13.455	**4.14**	C15H22	**benzene, 1-(1,5-dimethyl-4-hexenyl)-4-methyl-**
4	14.217	14.090	14.495	**6.82**	C15H24	**gamma.-curcumene**
5	17.548	17.485	17.600	0.94	C15H22O	beta.-Elemenone (CAS)
6	17.636	17.600	17.765	0.76	C9H14N2O3S	n-(4-hydroxyphenyl)-n,n',n'-trimethylsulfamide
7	18.615	18.535	18.630	0.11	C15H22O2	6-(1-Hydroxymethylvinyl)-4,8a-dimethyl-3,5,6,7,8,8a-hexahydro-1H-naphthalen-2-one
8	18.666	18.630	18.750	0.27	C17H33Cl	7-Heptadecene, 1-chloro- (CAS)
9	19.744	19.710	19.780	0.12	C15H26O	Juniper camphor
10	20.470	20.415	20.505	0.10	C24H32O6	medrol acetate
11	20.550	20.505	20.705	**3.04**	C9H12O	Cc1cc(C)c(O)c(C)c1
12	21.584	21.550	21.640	0.08	C20H40O	3,7,11,15-Tetramethyl-2-hexadecen-1-ol
13	21.922	21.880	22.010	0.13	C12H22O	2-methyl-10-undecenal
14	22.149	22.110	22.205	0.12	C21H38O4	9-Octadecenoic acid, 12-(acetyloxy)-, methyl ester, [R-(Z)]-(CAS)
15	22.745	22.695	22.785	0.08	C12H22O2	cyclooctancarbonic acid, 4-methyl-, ethylester
16	22.830	22.785	22.860	**3.36**	**C18H36O2**	Hexadecanoic acid, ethyl ester (CAS)
17	22.939	22.860	23.105	**16.49**	**C16H32O2**	Hexadecanoic acid (CAS)
18	23.135	23.105	23.365	**2.06**	**C20H40O2**	Eicosanoic acid (CAS)
19	23.864	23.795	23.895	0.24	C19H36O2	9-Octadecenoic acid, methyl ester (CAS)
20	23.915	23.895	23.975	0.10	C46H58N4O8	14'-epi-20'-deoxyvincovaline
21	24.410	24.345	24.465	**10.99**	**C20H36O2**	**Ethyl linoleate**
22	24.558	24.465	24.660	**33.65**	**C16H30O2**	**9-Hexadecenoic acid (CAS)**
23	24.689	24.660	24.925	**8.88**	**C18H36O2**	**octadecanoic acid**
24	25.014	24.925	25.110	**2.08**	**C22H38O2**	Cyclopropaneoctanoic acid, 2-[[2-[(2-ethylcyclopropyl)methyl]cyclopropyl]methyl]-, methyl ester (CAS)
25	25.136	25.110	25.170	0.56	C16H27NO4	6-Nitro-cylohexadecane-1,3-dione
26	25.224	25.170	25.265	**1.06**	**C18H36O3**	Hexadecanoic acid, 2-hydroxyethyl ester (CAS)
27	25.335	25.265	25.485	**1.12**	**C21H38O2**	11,14-Eicosadienoic acid, methyl ester (CAS)
28	26.151	26.110	26.200	0.10	C18H32O2	9,12-Octadecadienoic acid (Z,Z)- (CAS)
29	26.239	26.200	26.360	0.18	C18H34O2	9-Octadecenoic acid (Z)- (CAS)
30	26.426	26.360	26.505	0.27	C20H40O2	Eicosanoic acid (CAS)
31	26.680	26.640	26.720	0.10	C20H37ClO2	2-Chloroethyl oleate
32	26.943	26.885	27.055	0.69	C21H40O3	Oleic acid, 3-hydroxypropyl ester (CAS)
33	27.763	27.680	27.875	0.35	C19H38O4	Hexadecanoic acid, 2-hydroxy-1-(hydroxymethyl)ethyl ester (CAS)
34	28.855	28.780	28.895	0.09	C12H24O2	Decanoic acid, ethyl ester (CAS)
35	30.540	30.435	30.640	0.28	C20H38O3	9-Octadecenoic acid (Z)-, 2-hydroxyethyl ester (CAS)
	Total			100.00		

Supplementary material 4

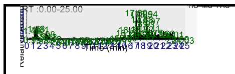

Figure 3. The total ion chromatogram of the CXBCH preparation was observed using LC-HRMS

Supplementary material 5

Tabel 3. Formula, molecule name, RT, annotation delta mass and max area (absolute) observed with LC-HRMS from CXBCH preparations.

No	Name	Formula	Annot. DeltaMass [ppm]	Calc. MW	RT [min]	Area (Max.)
1	Linoleic acid	C18 H32 O2	-3.41	280.239 27	22.71 3	7366556 9215
2	eremanthin	C15 H18 O2	-3.27	230.129 93	17.86 9	4914285 9119
3	1,5-Anhydro-D-fructose	C6 H10 O5	-2.94	162.052 35	1.57	2307692 8966
4	Oleic acid	C18 H34 O2	-3.18	282.254 98	20.52 7	2074895 4114
5	1,5-Anhydro-6-deoxy-D-threo-hex-1-en-3-ulose	C6 H8 O4	-2.08	144.041 96	1.606	1683113 5065
6	1-Linoleoyl glycerol	C21 H38 O4	-4.96	354.275 25	21.60 1	1612365 5143
7	Monoolein	C21 H40 O4	-4.44	356.291 08	23.05 8	1557725 1606
8	TURMERONE, AR-	C15 H20 O	-3.22	216.150 72	17.51 6	1029710 7430
9	Meglutol	C6 H10 O5	-2.54	162.052 41	3.029	1026964 4874
10	L-α-PALMITIN	C19 H38 O4	-4.13	330.275 64	22.52 8	9837543 783
11	9-Oxo-10(E),12(E)-octadecadienoic acid	C18 H30 O3	-3.01	294.218 61	19.10 8	9468689 328
12	(E)-6-hydroxyoctadec-4-enoic acid	C18 H34 O3	-3.02	298.249 9	19.44 4	9449669 222
13	Curcumin	C21 H20 O6	-3.55	368.124 68	16.26 6	9142424 073
14	(E,E)-alpha-Farnesene	C15 H24	-3.33	204.187 12	19.74 7	8176647 345

15	S-Curcumene	C15 H22	-3.69	202.171 4	18.93 8	7151614 798
16	9(Z),11(E),13(E)-Octadecatrienoic Acid methyl ester	C19 H32 O2	-3.8	292.239 12	20.09 3	6795616 369
17	Pyrogallol	C6 H6 O3	-1.7	126.031 48	3.29	6146490 443
18	(2E)-3-(3-Hydroxyphenyl)acrylaldehyde	C9 H8 O2	-2.26	148.052 1	17.86 1	4610742 991
19	11(Z),14(Z)-Eicosadienoic acid	C20 H36 O2	-2.98	308.270 61	21.89 7	4532720 559
20	(-)-Camphor	C10 H16 O	-2.9	152.119 67	15.39 5	4482468 927
21	α-Eleostearic acid	C18 H30 O2	-2.41	278.223 91	19.15 1	4016574 114
22	NP-020521	C18 H32 O3	-2.87	296.234 29	19.14 8	3964161 053
23	Prednisone	C21 H26 O5	-4.41	358.176 45	16.46 4	3457086 374
24	(2S)-2,3-Dihydroxypropyl (11Z,14Z)-11,14-icosadienoate	C23 H42 O4	-3.37	382.307 02	19.25 2	3356497 775
25	2,2'-Methylenebis(4-methyl-6-tert-butylphenol)	C23 H32 O2	-3	340.239 21	21.28 2	2849183 680
26	Carvone	C10 H14 O	-2.35	150.104 11	18.92 1	2780665 813
27	3,4-Dihydrocadalene	C15 H20	-3.23	200.155 86	19.10 5	2407722 995
28	Oleamide	C18 H35 N O	-2.8	281.271 08	22.39 9	2172824 383
29	Monoolein	C21 H40 O4	-4.45	356.291 07	22.48 2	1982732 023
30	1-Stearoylglycerol	C21 H42 O4	-4.28	358.306 77	20.71 6	1962687 838
31	Etretinate	C23 H30 O3	-2.87	354.218 48	19.74 6	1948205 351

32	NP-019983	C20 H16 O6	-3.39	352.0935	14.726	1934173852
33	Phloroglucinol	C6 H6 O3	-1.55	126.0315	3.555	1931287884
34	Stearidonic acid	C18 H28 O2	-2.32	276.20829	19.074	1815011551
36	geranyl quinone	C16 H20 O2	-2.98	244.1456	17.468	1656123930
37	5,6,7-Trihydroxy-8-(3-methyl-2-buten-1-yl)-4-phenyl-2H-chromen-2-one	C20 H18 O5	-2.99	338.11441	16.285	1552047892
38	5-hydroxy-4-methoxy-5,6-dihydro-2H-pyran-2-one	C6 H8 O4	-2.47	144.0419	3.556	1504039499
39	Wighteone	C20 H18 O5	-2.6	338.11454	16.636	1313576685
40	(2S)-3-(4-Hydroxyphenyl)-2-({[(3S,4S,5R)-2,3,4-trihydroxy-5-(hydroxymethyl)tetrahydro-2-furanyl]methyl}amino)propanoic acid (non-preferred name)	C15 H21 N O8	-3.71	343.12544	1.374	1253477230
41	8Z,11Z,14Z-Eicosatrienoic acid	C20 H34 O2	-3.9	306.25469	20.643	1239989132
42	(+/-)12(13)-DiHOME	C18 H34 O4	-2.72	314.24486	18.138	1175250981
43	NP-011548	C18 H34 O3	-3.44	298.24977	20.563	1122064288
44	α-Linolenic acid	C18 H30 O2	-2.95	278.22376	21.667	1080906094
45	(-)-Caryophyllene oxide	C15 H24 O	-3.36	220.18197	14.471	996365054.1
46	Curcumene	C15 H22	-3.69	202.1714	17.401	969170527.2
47	α-Eleostearic acid	C18 H30 O2	-2.37	278.22392	22.493	873227611.3
48	2,4-Dimethylbenzaldehyde	C9 H10 O	-2.78	134.07279	19.355	787523527.3
49	(3beta,24R,24'R)-fucosterol epoxide	C29 H48 O2	-3.84	428.36379	20.393	594526484.4

50	O-Demethylcurcumin	C20 H18 O6	-2.69	354.10938	15.868	581096744.5
51	(16beta)-16,21-Epoxypregna-4,17-diene-3,11,21-trione	C21 H24 O4	-3.32	340.16633	18.569	538639359.5
52	1-[(11Z,14Z)]-icosadienoyl-sn-glycero-3-phosphocholine	C28 H54 N O7 P	-3.18	547.36205	18.924	506081386
53	(+/-)9-HpODE	C18 H32 O4	-3.04	312.22911	18.056	463065694.7
54	16-Hydroxyhexadecanoic acid	C16 H32 O3	-2.33	272.23451	21.832	446057299.6
55	(15Z)-9,12,13-Trihydroxy-15-octadecenoic acid	C18 H34 O5	-2.25	330.23988	16.179	432687355.6
56	5-Methoxy-7-(4-hydroxy-3-methoxyphenyl)-1-phenyl-3-heptanone	C21 H26 O4	-3.17	342.18202	19.464	432029934.4
57	3-Ethylphenol	C8 H10 O	-2.9	122.07281	16.24	385526516.2
58	1,3,7-Trihydroxy-2,8-bis(3-methyl-2-buten-1-yl)-9H-xanthen-9-one	C23 H24 O5	4.96	380.16426	22.711	367961642.2
59	1-Naphthol	C10 H8 O	-2.35	144.05718	17.615	360952978.5
60	(±)9-HpODE	C18 H32 O4	-3.04	312.22911	19.356	339500663.4
61	all-trans-4,4'-diapo-zeta-carotene	C30 H44	-2.68	404.34322	18.901	335097027.5
62	1-Methyl-4-(1-methyl-2-propenyl)-benzene	C13 H18	-1.55	174.14058	18.947	325508927.1
63	Vanillin	C8 H8 O3	-1.47	152.04712	9.205	314499536.3
64	2-Ethyltoluene	C9 H12	-1.36	120.09374	19.361	302959828.6
65	citraurin	C30 H40 O2	-3.05	432.30151	22.046	265424339.3
66	13(S)-HOTrE	C18 H30 O3	-2.07	294.21889	18.658	253306661.7

67	Tapentadol	C14 H23 N O	-2.48	221.177 42	11.72 7	2360887 94.5
68	(8Z,11Z,14Z)-heptadecatrienoic acid	C17 H28 O2	-2.16	264.208 36	20.12 4	2299076 37.4
69	3,6-Anhydro-1-O-palmitoylhexitol	C22 H42 O6	-2.8	402.297 01	23.00 7	2212833 35.7
70	Linoleoyl ethanolamide	C20 H37 N O2	-4.16	323.281 08	17.41 6	2131231 16.3
71	Ferulic acid	C10 H10 O4	-4.12	194.057 11	10.48 6	2067817 09.5
72	5-Pentylresorcinol	C11 H16 O2	-2.77	180.114 53	13.83 6	2025544 56.5
73	9(Z),11(E),13(E)-Octadecatrienoic Acid methyl ester	C19 H32 O2	-3.78	292.239 13	20.95 4	1983259 31.6
74	(11E)-15-Oxo-11-icosenoic acid	C20 H36 O3	-3.31	324.265 37	21.92 4	1923538 81.8
75	1-(7,8-Dimethoxy-2,2-dimethyl-2H-chromen-6-yl)ethanone	C15 H18 O4	-2.79	262.119 78	17.02 6	1902883 45.4
76	8-Hydroxy-5,8a-dimethyl-3-methylene-3a,4,4a,7,8,8a,9,9a-octahydronaphtho[2,3-b]furan-2(3H)-one	C15 H20 O3	-3.04	248.140 49	18.39	1892165 10
77	1-Stearoyl-2-hydroxy-sn-glycero-3-PE	C23 H48 N O7 P	2.24	481.317 92	21.91 4	1729003 35.8
78	12-oxo Phytodienoic Acid	C18 H28 O3	-2.51	292.203 11	16.93 5	1514229 30.9
79	(2R)-3-Hydroxy-2-[(9Z,12E)-9,12-octadecadienoyloxy]propyl 2-(trimethylammonio)ethyl phosphate	C26 H50 N O7 P	-2.74	519.331 06	21.04	1361617 00.3
80	DL-carvone	C10 H14 O	-2.35	150.104 11	13.72 3	1350441 05.6
81	9(S)-HpOTrE	C18 H30 O4	-1.9	310.213 82	16.95 4	1271327 71.3
82	(15Z)-9,12,13-Trihydroxy-15-octadecenoic acid	C18 H34 O5	-2.25	330.239 88	16.71 1	1231487 09.5

83	3-oxopalmitic acid	C16 H30 O3	-2.37	270.218 85	20.85 7	1148304 50.8
84	6-Hydroxy-2,3,4-trimethoxybenzoic acid	C10 H12 O6	-2.35	228.062 85	1.482	1142056 34.8
85	2(N)-Methyl-norsalsolinol	C10 H13 N O2	-2.13	179.094 25	9.838	8962733 3.47
86	4-methoxy-6-(prop-2-en-1-yl)-2H-1,3-benzodioxole	C11 H12 O3	-2.14	192.078 23	13.07 2	8738365 7.19
87	Nicotinic acid	C6 H5 N O2	-2.03	123.031 78	21.42 2	8200516 6.33
88	(9S,10S)-10-Hydroxy-9-(phosphonooxy)octadecanoic acid	C18 H37 O7 P	-3.27	396.226 39	20.03	7509159 6.97
89	NP-015687	C19 H16 O4	-2.73	308.104 02	16.56	7378551 6.74
90	Citral	C10 H16 O	-2.98	152.119 66	17.56 5	7078606 1.68
91	Methylstyrylpyron	C14 H12 O4	-2.6	244.072 92	16.71	7035768 5.76
92	2,3,4,9-Tetrahydro-1H-β-carboline-3-carboxylic acid	C12 H12 N2 O2	-1.91	216.089 46	7.296	6222263 3.61
93	(2S)-2,3-Dihydroxypropyl (9Z)-9-hexadecenoate	C19 H36 O4	-3.42	328.260 24	19.81 9	6179148 1.82
94	(+)-(S)-Carvone	C10 H14 O	-2.35	150.104 11	11.27 5	6153951 8.31
95	1-Methyl-1,2,3,4-tetrahydro-Î²-carboline-3-carboxylic acid	C13 H14 N2 O2	-3.78	230.104 66	8.362	5275379 7.35
96	cis-12-Octadecenoic acid methyl ester	C19 H36 O2	-3.7	296.270 43	20.54 5	5150668 3.22
97	m-Cresol	C7 H8 O	0.05	108.057 52	17.86 9	5115550 4.38
98	(2E,4E,7E)-2,4,7-Decatrienoic acid	C10 H14 O2	-2.37	166.098 99	10.70 9	5107034 4.43
99	Ethyl oleate	C20 H38 O2	-4.8	310.285 69	22.24 1	5046085 1.23

100	Apocynin	C9 H10 O3	-2.23	166.06262	12.914	49615214.04
101	(-)-isopiperitenone	C10 H14 O	-2.46	150.1041	11.494	48182112.4
102	NP-003672	C15 H14 O2	-2.89	226.09873	14.555	46742344.31
103	Ibuprofen	C13 H18 O2	-2.29	206.13021	12.956	45378670.95
104	4-(4-hydroxy-2-methoxy-3,5,6-trimethylbenzoyloxy)-2-methoxy-3,5,6-trimethylbenzoicacid	C22 H26 O7	-2.93	402.16667	13.063	40793982.98
105	Quercetin	C15 H10 O7	-2.64	302.04185	13.547	39320299.41
106	3,4-Dihydroxycinnamaldehyde	C9 H8 O3	-2.2	164.04698	14.035	39215830.21
107	N-[(2E)-3-(3,4-Dihydroxyphenyl)-2-propenoyl]tryptophan	C20 H18 N2 O5	-4.69	366.11986	22.532	38726850.42
108	Palmiticacid	C16 H32 O2	-2.97	256.23947	21.612	38351961.64
109	Corchorifatty acid F	C18 H32 O5	-2.37	328.2242	15.449	36757398.95
110	4-Ethylguaiacol	C9 H12 O2	-2.23	152.08339	14.911	32907408.94
111	trans-10-Heptadecenoicacid	C17 H32 O2	-2.66	268.23952	17.861	32205740
112	3-dehydro-6-deoxoteasterone	C28 H48 O3	-4.18	432.35854	21.731	30837864.78
113	geranyl quinone	C16 H20 O2	-2.98	244.1456	22.518	30206293.08
114	NP-015559	C17 H14 O7	-3.03	330.07295	15.186	27277921.65
115	(10S)-Juvenile hormone III acid diol	C15 H26 O4	-2.79	270.18236	14.934	24724986.51
116	4-Coumaric acid	C9 H8 O3	-1.73	164.04706	10.038	24696560.89

117	10,16-Dihydroxyhexadecanoic acid	C16 H32 O4	-2.45	288.22935	17.975	20103158.23
118	COSMENE	C10 H14	-1.64	134.10933	11.29	18817198.48
119	3-Dimethylallyl-4-hydroxymandelic acid	C13 H16 O4	-3.89	236.10394	13.879	18473927.57
120	Levallorphan	C19 H25 N O	-1.7	283.19313	11.499	18236209.55
121	12-HSA	C18 H36 O3	-3.19	300.26549	17.292	17713187.6
122	(2Z)-2-(4-Hydroxybenzylidene)heptanoic acid	C14 H18 O3	-0.1	234.12557	15.182	17317207.37
123	Vitamin A	C20 H30 O	-2.89	286.22884	20.765	16961516.47
124	coenzyme Q2	C19 H26 O4	-3.9	318.18187	18.49	15080090.56
125	Octyl gallate	C15 H22 O5	-2.64	282.14598	14.471	14564065.2
126	4-Methoxycinnamaldehyde	C10 H10 O2	-2.5	162.06767	10.771	14494446.9
127	Guaiacol	C7 H8 O2	-0.66	124.05235	9.207	14343201.65
128	Methyl linoleate	C19 H34 O2	-3.97	294.25471	20.686	14211480.42
129	Eicosapentanoic acid	C20 H30 O2	-3.49	302.22352	22.58	14208703.13
130	Nandrolone	C18 H26 O2	-2.76	274.19252	14.807	13243367.08
131	(4E)-1-(4-Hydroxyphenyl)-4-decen-3-one	C16 H22 O2	-3	246.16124	16.037	12478489.91
132	2-Methoxyresorcinol	C7 H8 O3	-3.01	140.04692	9.645	12411623.58
133	7-Hydroxyflavan	C15 H14 O2	-2.89	226.09873	11.653	11359843.24

134	4,8,12-trimethyltrideca 1,3,7,11-tetraene	C16 H26	-2.81	218.20284	20.816	9897947.401
135	Eicosapentanoic acid	C20 H30 O2	-3.49	302.22352	22.173	8676767.851
136	3-Methyl-2-butenyl caffeate	C14 H16 O4	-1.47	248.10449	13.214	8511089.247
137	Thymoquinone	C10 H12 O2	-1.6	164.08347	12.505	8440170.481
138	Isorhamnetin	C16 H12 O7	-2.22	316.0576	14.843	8058961.505
139	2-Octenoic acid	C8 H14 O2	-1.16	142.09921	10.807	7572010.605
140	1-(3,4-Dihydroxyphenyl)acetone	C9 H10 O3	-2.23	166.06262	10.76	6400114.457

Supplementary material 6

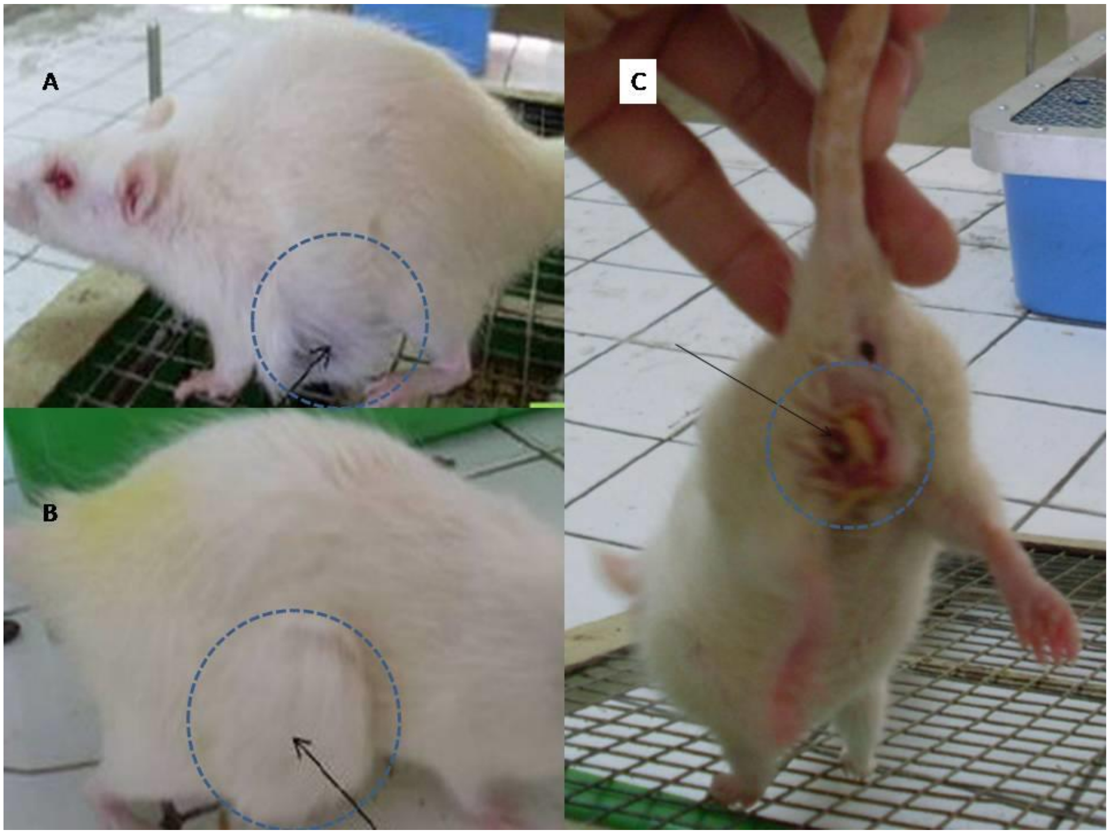

Figure 8. Nodules (arrows) were formed in the mammary gland in the 20th week of observation of SD rats after receiving CXBCH 2 weeks before and five weeks after DMBA induction. Nodules formed in all parts of the mammary gland (fore legs (A), near the hind legs (B), and some nodules break up (C). Note: Arrows indicate nodules
